# The Understanding Capacity and Information Dynamics in the Human Brain

**DOI:** 10.3390/e21030308

**Published:** 2019-03-21

**Authors:** Yan M. Yufik

**Affiliations:** Virtual Structures Research, Inc., Potomac, MD 20854, USA; imc.yufik@att.net

**Keywords:** understanding, mental models, information production, negentropy generation

## Abstract

This article proposes a theory of neuronal processes underlying cognition, focusing on the mechanisms of understanding in the human brain. Understanding is a product of mental modeling. The paper argues that mental modeling is a form of information production inside the neuronal system extending the reach of human cognition “beyond the information given” (Bruner, J.S., *Beyond the Information Given*, 1973). Mental modeling enables forms of learning and prediction (learning with understanding and prediction via explanation) that are unique to humans, allowing robust performance under unfamiliar conditions having no precedents in the past history. The proposed theory centers on the notions of self-organization and emergent properties of collective behavior in the neuronal substrate. The theory motivates new approaches in the design of intelligent artifacts (machine understanding) that are complementary to those underlying the technology of machine learning.

## 1. Preview

The article presents a theory of understanding that boils down to the following. The brain is a regulatory system orchestrating sensory-motor responses to varying external conditions. Thermodynamics steers self-organization in the brain towards producing quasi-stable neuronal groupings (called packets) that can be re-combined rather than composed anew each time the conditions change. Packet combinations serve as models of the environment that can be exercised under the guidance of external feedback, or without such guidance (simulation, thinking). Self-directed construction and manipulation of mental models underlie understanding. Absorbing information via feedback constitutes negentropy extraction. Mental modeling in the absence of such feedback equates to producing information and generating negentropy. The modeling capability is a recent evolutionary discovery that yielded high regulatory efficiency, and at the same time, created a uniquely human need for understanding the world, which is related to, but distinct from, the need to organize interaction with the world in a manner conducive to survival. These suggestions will be unpacked in several iterations, starting with the remainder of this preview. 

This paper argues that a definitive feature of information dynamics in the human brain is a particular form of information production [1] that underlies the understanding capacity and is unique to the human species. Information is produced when regularities intrinsic to the processing system are superposed on regularities in the incoming signal (e.g., correlations in the stimuli stream). Perception is a form of information production shared by animals and humans. In perception, e.g., vision, correlated excitations of receptor cells in the retina are organized into images (mental objects) that are projected outside and experienced as physical objects located in the space beyond the receptor surface. Understanding involves construction of mental models that go beyond perception, in two ways. First, models represent not only objects but their changes over time (behavior) and the ways the changes are mutually coordinated (inter-object relations). Second, perception operates on sensory signals, while modeling (“thinking”) is decoupled from the sensory inflows. Constructing models and operating on them in the absence of motor-sensory feedback constitutes a uniquely human form of information production (see Section 5.2.4 for clarifications). 

Projecting the products of mental modeling into the outside world underlies the experience of having apprehended salient relations in the environment, which enables explanation, anticipation of changes, and planning actions likely to influence such changes in the desired direction. Understanding complements learning; learning re-uses responses found to be successful under circumstances similar to the present conditions. In contrast, mental modeling takes advantage of the past experiences without repeating them, by allowing re-combination in the construction of novel responses. In short, understanding enables construction of robust (near-optimal) responses to disruptive changes, and anticipatory preparation to possible changes before their onset. 

How exactly are the models constructed and exercised? These questions have not received much attention, and answers to them are unknown. This paper makes the following suggestions: mental models are constituted by neuronal structures acting as “synergistic wholes”, where changes in one component constrain changes in the other components. “Synergistic wholes” radically reduce the number of degrees of freedom in their components (spontaneous and attentive activities engage distinct neuronal systems, discussed in some detail in the concluding sections section). As a result, exercising a model, e.g., varying parameters in a particular component, produces variations throughout the structure within narrow ranges allowed by inter-component coordination. Formation of “synergistic wholes” is spontaneous, while parameter variations are amenable to attentive examination (Section 5.2.2 expands on that notion). Suppression of degrees of freedom and complexity collapse in mental models enable attentive reasoning by maintaining attentive processes within narrow bounds of parameter variations afforded by the model. Since attentive processes demand energy, the mental modeling capacity yields radical reductions in the demand. As a result, humans can handle complex coordination problems (e.g., managing battles, playing chess, designing complex artifacts, etc.) with small energy budgets. Formation of modeling hierarchies underlies the experience of growing understanding and gradual development of a coherent worldview, revealing unifying principles behind expanding multitudes of diverse phenomena. 

In the remainder of the paper, these suggestions will be elaborated following three lines of enquiry:

(1) Self-organization in the neuronal substrate;
(2) Information production; and
(3) Optimization of the organism–environment interaction.

Energy is a universal interaction currency. Accordingly, all three lines converge in the notion of thermodynamic efficiency. Mental modeling is a form of information production, which yields the dual benefits of minimizing energy expenditure inside the system, while maximizing energy inflows from the outside (hence, optimization). Information efficiency is a corollary of thermodynamic efficiency—minimal amount of sampling obtains maximally valuable information. Importantly, associating mental modeling with self-organization entails differentiation between extrinsic and intrinsic sources of value. Extrinsic values are determined by energy extraction opportunities that the information signifies, while intrinsic values (worth, significance) are determined by self-organization opportunities that the information enables. Intrinsic values motivate information seeking and production, experienced as unification of models and interlocking of modeling hierarchies. In this way, decoupling from sensory inputs gives rise to uniquely human pursuits, separating intellectual significance of information from the material benefits it can bring about.

The proposed theory relies on two notions: neuronal packets and virtual associative networks. Neuronal packets are Hebbian assemblies stabilized by boundary energy barriers. Formation of bounded, quasi-stable neuronal packets underlies perception, that is, gives rise to bounded, quasi-stable feature aggregations (images, mental objects) projected into the outside space. Packets form in associative networks capturing correlations in the sensory stream; strong correlations trigger phase transition in tightly associated neuronal subnets causing their segregation and folding into packets. Packets establish an energy landscape over the associative network, regulating attentional access to packet internals (high boundary barriers deny access or cause attention capture inside packets). Virtual network comprises a hierarchy of functional networks establishing coordinations (relations) between individual packets, between packet groupings, between groups of groups, etc. In this way, information assimilated in the form of associative links of varying strength (synaptic weights) gives rise to a self-produced functional hierarchy, transforming sensory flux into unifying world models of growing generality. As succinctly stated by Eddington, “the mind filters out matter from meaningless jumble of qualities, as the prism filters out the colors of the rainbow from the chaotic pulsation of white light” [2].

These ideas have motivated the following conjecture: Understanding boils down to apprehending coordination in the behavior of mental objects. Accordingly, the onset of the understanding and language capacities approximately 80,000 years ago (Cognitive Revolution [3]) could be the result of evolutionary advancement when the apparatus of sensory-motor coordination richly developed in the protohuman, optimized for manipulating external objects under the control of motor-sensory feedback, was co-opted for the manipulation of mental objects in the absence of such feedback. Similarly, the signaling system was co-opted to support the handling of mental objects. These conjectures will be addressed in the outline of the theory. 

Operationalizing these ideas motivated a computational architecture (dubbed “gnostron”) simulating some of the processes presumed to underlie human understanding. The gnostron approach is orthogonal to that implemented in perceptron, and picks up where the perceptron leaves off. Perceptron establishes a mosaic of synaptic weights and gnostron operates on that mosaic while leaving the weights intact. The concluding discussion will compare the two approaches.

To complete the preview, some limitations of the proposed theory need to be pointed out. In particular, the theory uses the notion of “mental models” in a restricted way and in a manner that does not always agree with the meaning attributed to this notion in the literature. According to Wikipedia, “a mental model is a kind of internal symbol or representation of external reality, hypothesized to play a major role in cognition, reasoning, and decision making … In psychology, the term mental models is sometimes used to refer to mental representations or mental simulation generally… scientific debate continues about whether human reasoning is based on mental models, versus formal rules of inference, or domain-specific rules of inference or probabilities”. As stated above, the present theory uses the term to denote structures comprised of neuronal packets and suggests that human understanding is rooted in operations based on such structures. It can be ascertained that those same operations are involved in problem solving, reasoning, inference, decision making, etc., but the paper does not enter the debate and recognizes that this theory in its present form might fail to account adequately for all aspects and nuances of these and other cognitive processes. Identifying such shortcomings in the present version of the theory is a necessary step for future work, which will lead to revisions and new synthesis. 

Besides mental modeling, the theory addresses other cognitive processes, such as attention and motivation. The article defines these processes within the framework of the theory and as the theory is outlined, postponing clarifications until the discussion section. The important task of comparing definitions and opinions in this paper with the plethora of definitions and opinions in the literature is assigned to future research. Some key notions are repeated throughout the text, on the assumption that readers will tolerate some redundancy for the sake of clarity. 

The article is organized into five parts, including the introduction and concluding discussion. The introductory part seeks to place the theory of understanding within a broad framework, combining philosophy (the mind-matter dichotomy), information theory, thermodynamics (self-organization in open, far-from-equilibrium systems), and optimal control. The introduction concludes by formulating “principles of understanding”. Part 3 outlines the proposed theory, and summarizes theoretical results and experimental findings to date that appear to corroborate it. Part 4 applies the theory to address topics overlapping with that of understanding, such as consciousness, mental modeling in norms, and pathology, among other. The theory affords treatment of these complex topics that is exceedingly simple and coheres with insights expressed in some other theories. The lines of treatment are only tentatively stated, in the hope of motivating other researchers to pursue them further. The concluding part 5 summarizes the proposal and makes suggestions for further research.

## 2. Introduction

This part consults philosophy, seeking actionable ideas about the nature of intelligence and the role of understanding. The findings are condensed into “principles of understanding”, setting the stage for a theory of understanding outlined in the next part. 

### 2.1. The Physical and the Mental

In the following definition, introduced by Karl Popper, understanding operates in “the world of the products of our human minds” [4], or World 3 built on top of World 1 (conditions in the environment) and World 2 (representations of external conditions in the brain) [4,5]. The following section takes a closer look at the three Worlds, seeking to define the role of operations in World 3 in overcoming challenges encountered in World 1. 

Arguably, the intuition of three Worlds [4,5] was already implicit in Aristotle’s Law of Identity, as follows. The law states that each thing (object) *A* is identical to itself,

(∀A) (A=A),

which can be interpreted as addressing identity preservation in:

(a) Material (physical) objects $A_{p}$ = $A_{p}$;
(b) Mental objects $A_{m}$ = $A_{m}$;
(c) Relationships between the material and the mental objects, responsible for making the former accessible to the latter (i.e., intelligible) $A_{p}⇆A_{m}$.

The remainder of the section examines these interpretations in order to derive intelligibility requirements as conditions under which physical objects get represented in the mental domain $A_{p\;}\to A_{m}$ in a manner allowing operations in the mental domain to guide actions in the physical domain $A_{m}\to A_{p}$.

#### 2.1.1. Physical Objects

Physics endows objects with three definitive attributes—they are distinct, persevere in their self-identity, and can interact with other objects, as follows. Firstly, two objects can be identical but distinct; for example, two copies of the same book fresh from the printer. Secondly, objects can behave; that is, change their properties and sustain a certain amount of such changes without losing their self-identity (e.g., a copy of a book that is worn out and marked all over can remain self-identical. Self-identity is not violated when a book is moved or placed inside a container, but can be lost when the book is torn apart, etc.). Thirdly, objects can have relations with other objects, imposing different forms of behavior coordination (e.g., placing a book in a briefcase enforces a particular form of behavior coordination between them). 

A reassessment of the notion of “material objects” in modern physics led to the realization that objects can be of two types, admitting different forms of properties and relationships, as follows. A common sense view (Newtonian physics) endows objects with distinct spatial boundaries and positions in space so that the volume occupied by an object cannot admit other objects. Also, transferring objects between volumes is possible only via continuous movement. This view does not hold onto the micro scale: micro objects neither have distinct spatial boundaries and positions in space, nor move continuously. Coordination between macro objects requires either a direct contact or some intermediaries that convey force or information. By contrast, on the micro scale, coordination can occur across any distance and without intermediaries (i.e., entanglement). Finally, properties of micro objects cannot be decoupled from those of the measuring device. To generalize, it can be said that material objects have attributes conforming to Newtonian or quantum-theoretic approximations. 

#### 2.1.2. Mental Objects

Mental objects are the “contents being attended to in consciousness” (The Harper Collins Dictionary of Philosophy, 1992). To satisfy the $A_{p}\;⇄\;A_{m}$ requirement, mental objects must, as a minimum, represent the definitive attributes of their physical counterparts. They must be amenable to reliable attentive discrimination, must have a degree of stability, and must combine stability with flexibility to allow representing changes in the objects without compromising their self-identity. Representing interactions between physical objects requires reversibility of mental operations, that is, the ability to juxtapose mental entities $A_{m}^{1}$, $A_{m}^{2}$, …, $A_{m}^{n}$, and shift attention between them without distorting the entities [6,7].

#### 2.1.3. Requirements for Intelligibility

The Law of Identity appears to assert that:

1. The physical world is intelligible because it has a degree of order and consistency;
2. Processes in the brain serve to apprehend that order and apply the results in regulating behavior;
3. Efficient regulation is predicated on the availability of reversible operations performed on distinct (segregated), (quasi)stable, and flexible informational structures (mental objects).

### 2.2. Evolution of Regulatory Mechanisms

Life emerges in networks of interacting units (e.g., complex molecules), when subnetworks fold into structures bounded by Markov blankets (separating surfaces, or “membranes” [8]) that preserve connectivity between the subnet and its surrounds, while, at the same time, according to the subnet, a degree of statistical independence [9,10,11]). Life is sustained via flows of energy and matter through the “blanket”, including an influx of high-grade energy and expulsion of degraded energy (heat) and waste. Accordingly, emergence of life must be concomitant with formation of flow regulating mechanisms, which can subsequently evolve towards increasing regulatory efficiency. Roughly, three evolutionary stages can be identified, as follows:

(A) Primitive organisms. Regulation is confined to the boundary surface (e.g., opening or closing surface channels to allow or block access to the organism’s internals).
(B) Animals. Regulation expands to the immediate surrounds and manifests in a range of behaviors extending from simple reactions (e.g., sea slugs extending or withdrawing their gills) to complex predatory or foraging behaviors in higher animals. In all cases, acting on conditions external to the surface involves establishing direct contact with the surface (reaching, grabbing, clawing, biting, etc.).
(C) Humans. Regulation expands to outside conditions separated from the organism’s boundary surface by indefinitely large intervals. As formulated by Bertrand Russell: “…the essential practical function of “consciousness” and “thought” is that they enable us to act with reference to what is distant in time and space, although it is not at present stimulating our senses” [12].

Since separation in space and time prevent acting on the objects directly, regulation must incorporate mechanisms allowing coordinated engagement of multiple intermediary objects (e.g., tools). It will be argued that: (a) anticipatory construction of such coordinations, decoupled from the current sensory inputs and overt actions, is the essence of understanding and the subject of “thinking”; (b) language and explanation capabilities are components of understanding, enabling construction of indefinitely expandable coordination hierarchies; and (c) construction of coordinations (mental modeling) is a form of information production. 

Anticipatory construction allows organisms to prepare for the forthcoming events before their onset. In that sense, the function of intelligence includes curbing surprise. Accordingly, a general principle of brain operation can be expressed in terms of surprise minimization (more technically, Minimization of Variational Free Energy (MVFE), determined by the amplitude of discrepancies between the anticipated events and those eventually encountered [11]). Understanding reduces or preempts surprise, and thus conforms to this general principle (part 3 suggests some extensions of the MVFE principle motivated by the theory of understanding). 

Self-directed construction of mental models is a form of self-organization. Accordingly, formulating a theory of understanding requires elucidating biophysical mechanisms of self-organization in the neuronal substrate. These mechanisms are presently unknown, although their general characteristics can be ascertained, leading to principles of understanding. Some principles are suggested in the next section, prefaced by a brief excursion into biophysics. 

### 2.3. Principles of Understanding

Reversibility of mental operations [6,7] demands low entropy and imposes other constraints on the thermodynamic mechanisms operating in the human brain (simply stated, there can be no thinking if one cannot return to the contents of earlier thoughts, because the contents or the pathways leading to them have eroded and are no longer available). More precisely, mental operations underlying understanding in adults were shown to acquire the properties of algebraic groups, which includes capacity for re-visiting mental objects and combining objects into functional associative groupings [6,7,12]. Consistent with these findings and suggestions in Section 2.1, it can be surmised that the neuronal substrate of understanding must allow formation of quasi-stable neuronal aggregations that are capable of withstanding entropic erosion and are amenable to grouping into composite structures (i.e., mental models), entailing progressive entropy reduction and a growing degree of organization in the neuronal system. This section lists some of the results in the literature, shedding light on the mechanisms of self-organization and entropy reduction.

(1) Self-organization feeds on energy. More precisely, “the flow of energy through a system acts to organize that system” [13]. Entropy *H* of an open system ϕ connected to an energy source and energy sink is determined by entropy of the system $H_{\phi }$ and cumulative entropy of the source and the sink $H_{ss}$, *H* = $H_{\phi }$ +$H_{ss}$. According to the second law, $dH_{\phi }$ + $dH_{ss}$ ≫ 0. Energy flow from the source to the sink leads to increasing entropy in the source-sink subsystem, $dH_{ss}$ > 0. The only demand on entropy change in ϕ placed by the second law is that $-dH_{\phi }$ $\ll \;dH_{ss}$. Accordingly, entropy decreases in ϕ are permitted, under the condition that system ϕ is open and serves as a conduit for energy flow [13].
(2) Self-organization takes place in open systems driven away from equilibrium (“dissipative systems”) [14,15], and proceeds through phase transitions accompanied by entropy reduction and symmetry changes [16,17]. The rate of entropy generation declines as systems relax toward steady states [18]. Changes of symmetry manifest, for example, in the formation of Benard cells when molecular mechanisms of heat transfer are replaced with convective heat transfer. In general, symmetry breakings accompany transitions from disorganized movements of individual (micro) units to collective movement of ensembles comprised of multiple units (as, for example, during transitions from laminar to turbulent flow in liquids [19,20]).
(3) Information absorption entails entropy reduction and extraction of free energy [21,22,23,24]. The notion dates back to the realization that measurements yielding information *dI* about a physical system cause entropy increase inside that system [21]. Reciprocally, absorbing information *dI* reduces entropy *dH* in the receiver, accompanied by extraction of free energy *dF* and conversion of heat into work *dW*. Roughly, the argument is as follows [24].

**Figure 1:**
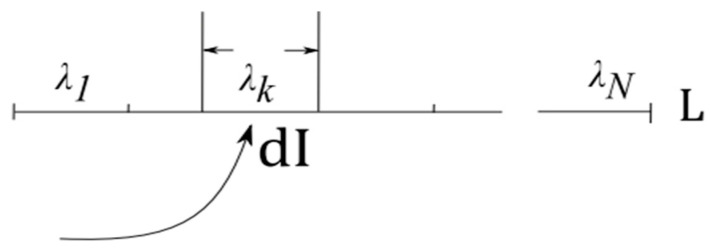
Absorbing information *dI* narrows the range of states available to system $\phi_{1}$ *(λ)* to domain $\lambda_{k}$ (adopted from [24]).

Assume that system $\phi_{1}$*(λ)*, *λ* ∈L is connected to a thermal bath at temperature *T* and is positioned to receive information from system $\phi_{2}$*(ξ)*, *ξ* ∈ *Ξ*. The initial state of $\phi_{1}$*(λ)* is defined by distribution *p(λ)* and entropy *H*, *p(λ) = exp((F(λ) − Ε(λ))/Τ),* where *F(λ)* and *E(λ)* denote free and total energy, correspondingly. Initially, $\mathrm{system}\;\phi_{1}$*(λ)* is uncertain about the state of $\phi_{2}$*(ξ)* (any value of *λ, λ* ∈L is possible), while absorbing information *dI* from $\phi_{2}$*(ξ)* would set *λ* to some $\lambda_{0}$, and thus eliminate the uncertainty (by indicating that $\phi_{2}$*(ξ)* is in some state *ξ* = $\xi_{0}$). Let information *dI* allow only partial uncertainty reduction and the corresponding entropy decrease *dH.* To assess the scope of these changes, partition *L* into *N* domains separated by “barriers,” as in Figure 1, and assume that *dI* confines $\phi_{1}$*(λ)* to domain $\lambda_{k}$ in *L*, with *F, E*, and *H* assuming posterior values *F(*$\lambda_{k}$*), E(*$\lambda_{k}$*)*, and *H(*$\lambda_{k}$*)*, respectively (temperature is kept constant during the process, by maintaining connection to the thermal bath). Restoring the initial distribution would require pushing the “barriers” apart until $\lambda_{k}$ = L. The movement is resisted by the environment and requires forces sufficient for overcoming the resistance. 

Energy for carrying out the requisite amount of work *dW* is drawn from the thermal bath, obtaining free energy increment *dF = F(*$\lambda_{k}$) − *F*. In this way, absorbing information enables conversion of thermal energy into the amount of work bounded by the product of entropy and information intake, dW≤H×dI (dW=H×dI if conversion proceeds slowly, t→∞ and dW<H×dI if otherwise). These conclusions allow re-formulation of the second law

(1) dH+dI≥0

Equation (1) indicates reduction of entropy in the receiving system following absorption of information, and conversion of thermal energy into work (or, indirectly, information into work) [24,25]. 

Analysis of relationships between information, free energy, work, and thermodynamic entropy has substantially advanced in the last decade (e.g., [26,27,28]). However, analysis in [24] was carried beyond the examination of these relationships, proposing that worth (significance) is attributed to information as a function of entropy decrement produced by information absorption; the larger the decrement, the higher the significance. Attributing significance to information expands Shannon’s information-theoretic framework [29] in a direction particularly relevant to the present proposal. Specifically, the relationship suggests that the human brain does not absorb whatever information comes its way but actively seeks information conducive to its progressive self-organization (these notions will be re-visited in the later sections). 

In summary, suggestions in the preceding sections and theoretical results referenced in this section can be summarized in several hypotheses concerning operation of understanding (the principles of understanding), as follows:

(1) Understanding is a product of self-organization in the neuronal substrate, involving self-directed construction and manipulation of mental models.
(2) Models are composed of quasi-stable neuronal groupings (packets).
(3) Mental modeling involves work and is predicated on supplying free energy in the amounts sufficient for performing that work. The human brain regulates extraction of free energy from the environment and diverts a part of it towards the work of mental modeling.
(4) Modeling produces information. Absorbing information from the environment equates to negentropy extraction, and reducing entropy as a result of internal information production equates to negentropy generation.
(5) The process is self-catalytic, in the sense that modeling stimulates information seeking on the outside (significant information) that facilitates increasing order on the inside, via formation of new models, and expanding and unifying the already formed models.
(6) Mental models function as synergistic complexes, focusing energy delivery and obtaining large amounts of work at low energy costs (high-cost attentive changes in any component produce mutually coordinated, low-cost changes throughout the model).
(7) Modeling yields a quantum leap in regulatory efficiency by improving energy extraction from the outside (better predictions and robust response construction in unforeseen circumstances) and reducing unproductive energy expenditures inside the system.

The next part outlines a theory substantiating these hypotheses.

## 3. Theory of Understanding: How the Intelligible World Arises from Sensory Flux

The theory has been presented elsewhere [30,31,32,33], while the following sections summarize some of the key ideas, in three iterations. First, principles 1–5 are addressed, focusing on the formation of neuronal groups and their role in understanding. Next, experimental findings are reviewed in neuroscience and psychology concerning the dynamics of neuronal groups and the operation of understanding. Finally, principles 6 and 7 are addressed, focusing on the benefits of understanding. 

### 3.1. Neuronal Packets and Their Role in Understanding

Understanding one’s environment involves constructing models capturing objects, their behavior, and the forms of behavior coordination (inter-object relations). The theory of understanding pivots on the idea of “neuronal packets.”

#### 3.1.1. Neuronal Packets: The Building Blocks of Understanding

**Figure 2:**
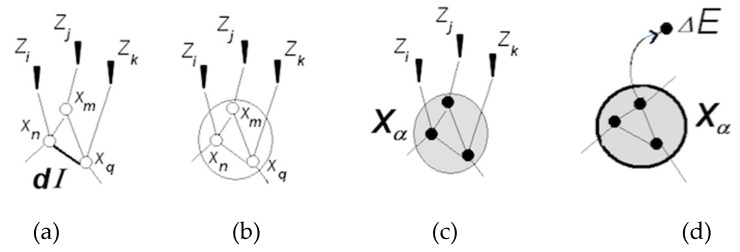
(**a**) Repetitive co-occurrence of stimuli $z_{i}$ and $z_{k}$ causes repetitive co-firing of neurons $x_{n}$ and $x_{q}$ and amplification of synaptic connections between them. As a result, the neuronal system receives information *dI* registered (absorbed) in the form of $x_{n}$–$x_{q}$ synaptic link. (**b**) Hebbian assembly emerges, as an aggregation of neurons that are tightly associated with each other due to their exposure to strongly correlated stimuli [34]. (**c**) Phase transition turns assembly into a quasi-stable packet $X_{\alpha }$ separated by boundary energy barrier from the surrounds. (**d**) Removing neurons from the packet requires expenditure of energy Δ*E* sufficient for overcoming the barrier.

Neuronal packets are Hebbian assemblies [34,35], or subnets that form in associative networks and are segregated from the surrounding network by boundary energy barriers. It was hypothesized that energy barriers emerge as a result of phase transition in tightly associated subnets. Energy barriers make the packets distinct and quasi-stable; that is, stable enough to sustain changes in the surrounds without erosion and flexible enough to allow internal changes responsive to the external ones. Barriers determine the extent of internal changes that packets can afford without losing their integrity. Figuratively, neuronal packets are blocks of which the edifice of the intelligible world is constructed [30,31]. Figure 2 illustrates stages in the formation of packets.

Arguably, the requirements of segregation, stability, and flexibility were implicit in the foundational idea of neuronal assembly [34]. Absent from such barriers, assemblies could exist only as transient aggregations experienced as sporadic sensory clusters flickering against the background of sensory flux (or “meaningless jumble of qualities” [2]). By contrast, bounded, quasi-stable packets give rise to the experience of bounded, quasi-stable objects populating the environment.

#### 3.1.2. Perception and Recognition

**Figure 3:**
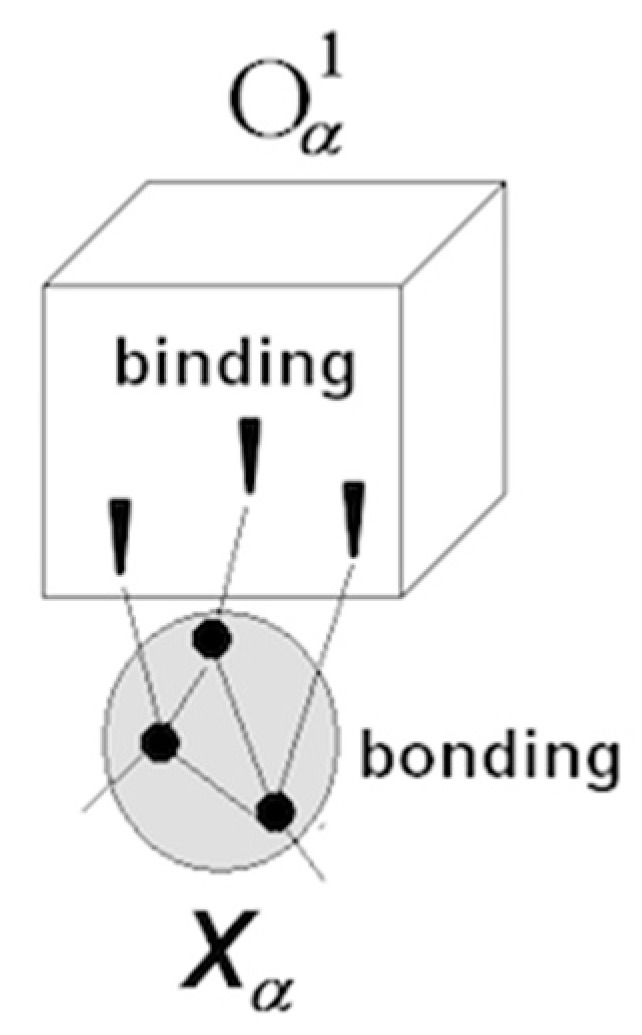
Emergence of packet $X_{\alpha }$ gives rise to the experience of object $O_{\alpha }$ projected into physical space outside the sensory surface (e.g., visual images are not experienced as irritations of the retina). Binding between object $O_{\alpha }$ and packet $X_{\alpha }$ is predicated on “bonding” in $X_{\alpha }$ (i.e., cohesion and relative stability of neuronal packets underlying the experience of external objects).

Perception of objects (bounded, quasi-stable feature aggregations) results from projecting neuronal packets into the outside space. Images are entities in the mental space that mediate interaction between entities in the neuronal space (neuronal packets) and their counterparts in the physical space (physical objects). Figure 3 illustrates these notions. 

**Figure 4:**
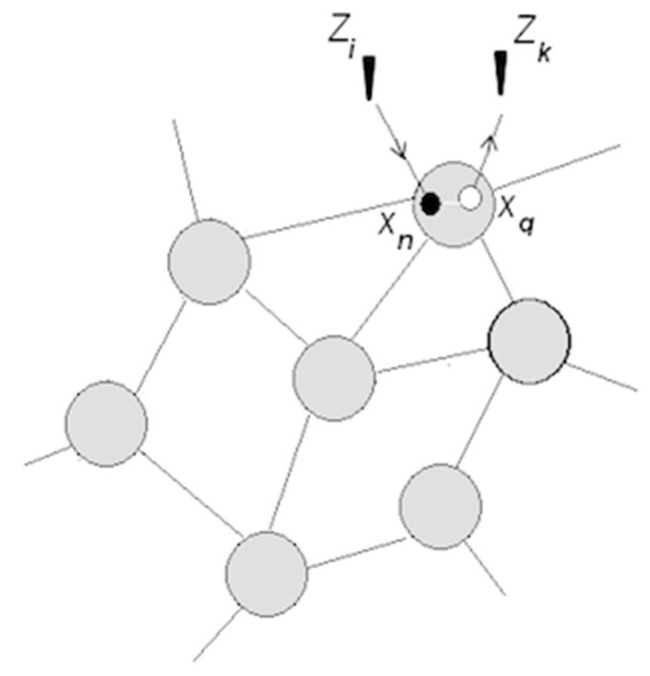
Packets partition neuronal space into “domains” separated by energy barriers (seen in Figure 1). Before the arrival of stimulus $z_{i}$, the system was uncertain about the state of the environment; stimulus $z_{i}$ “primes” one of the packets, i.e., confines choices to a particular “domain” (packet), and thus predicts the likely stimuli composition (e.g., stimulus $Z_{K}$). Firing $x_{q}$ confirms (or disconfirms) the prediction. A series of confirmations amounts to recognizing the object (see Figure 3), while failed prediction motivate shifts to other packets.

Packets form as a result of phase transition in associative networks causing emergence of functional surfaces separating packet internals from the surrounding network. Tendency towards free energy reduction in the surface creates bonding that holds the neurons together in quasi-stable aggregations (packets are not unlike raindrops, where molecules are held together by surface tension [32]). Waves of phase transition propagating in the associative network partition neuronal space and populate physical space with objects amenable to recognition, as illustrated in Figure 4. 

Packets represent objects in both Newtonian and quantum-theoretic approximations (representing spatially unbounded physical fields still requires a bounded mental structure (mental object) that is distinct and separable from other such objects, for example, maintaining distinction between the notions of an electric field and magnetic field).

#### 3.1.3. Apprehending Behavior

**Figure 5:**
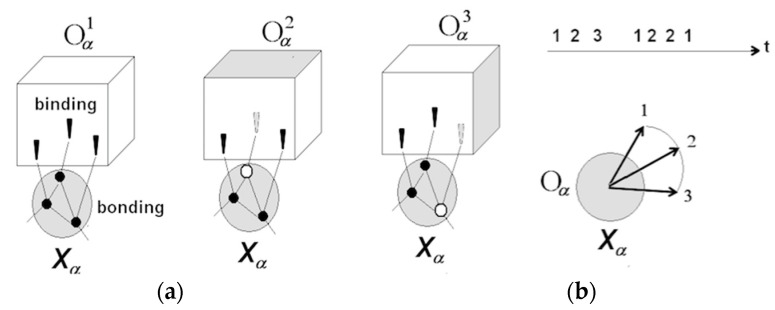
(**a**) Changing response patterns in packet $X_{\alpha }$ underlies the experience of object $O_{\alpha }$ undergoing a series of state changes $Q_{\alpha }^{1}\to Q_{\alpha }^{2}\to Q_{\alpha }^{3}$. The object preserves its self-identity due to the preserved $O_{\alpha }$ –$X_{\alpha }$ binding $(Q_{\alpha }^{1},\;\;Q_{\alpha }^{2},\;Q_{\alpha \;}^{3}$ are experienced as different states of object $O_{\alpha }$). (**b**) If population response vector is computed on the group of neurons in packet $X_{\alpha }$, changes in $O_{\alpha }$ can be represented as rotation of the population vector. Different rotation trajectories define the behavior repertoire available to the object.

Packets afford a range of variations in their response patterns without losing stability, giving rise to the experience of changes in the corresponding objects. Figure 5 explains the notion. 

As indicated in Figure 5b, switching rotation trajectories of packet vectors underlies the experience of having apprehended different behavior patterns available to object $O_{\alpha }$ ($Q_{\alpha }^{1}\to Q_{\alpha }^{2}\to Q_{\alpha }^{3}$, $Q_{\alpha }^{1}\to Q_{\alpha }^{2}\to Q_{\alpha }^{2}\;\to Q_{\alpha }^{1}$, etc.). Note that packets respond asynchronously. Synchronous firing equates to being able to recognize objects despite changes ($Q_{\alpha }^{1}\;\mathrm{is}\;\mathrm{the}\;\mathrm{same}\;\mathrm{as}\;Q_{\alpha }^{2},\;\mathrm{etc}.),$ while being unable to pinpoint those changes and apprehend their succession (object’s behavior). Recognition via synchronous firing gives a coarse-grain view of the environment and is energetically wasteful. 

To summarize, behavior of an object is the totality of changes the object affords. Packets respond asynchronously, representing changes in the object by different activation-inhibition patterns of the constituent neurons. As depicted in Figure 5, three neurons in a packet respond to three features of an object (conditions (dark or light) on one of the facets of a cube). Neuron is ON (excited) if the corresponding facet is light and OFF (inhibited) if otherwise. These thee neurons define packet vector rotation depending on the combination of conditions. Accordingly, a particular behavior (e.g., only the front facet is dark → only the top facet is dark → all three facets are dark → …) defines a particular trajectory of the packet vector movement. 

#### 3.1.4. Rudimentary Understanding

**Figure 6:**
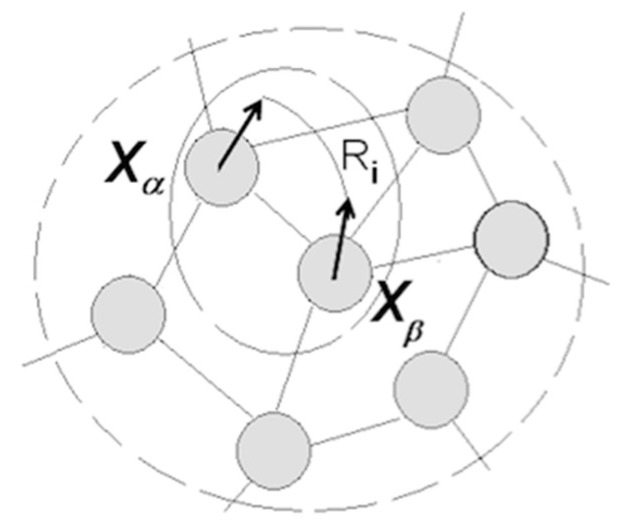
Link $R_{i}$ connecting packet vectors in adjacent packets $X_{\alpha }$ and $X_{\beta }\;$ denotes ability to rotate packet vectors in a coordinated fashion, and to detect coordinated rotation.

The next advancement in the evolution of intelligence involves emergence of a limited capability to apprehend behavior and manipulate mental objects in a coordinated fashion, to produce behavior changes leading towards the desired outcome. Figure 6 illustrates this advancement. 

Self-initiated coordinated rotation of several packet vectors manifests in purposeful manipulation of physical objects. In animals, the capability is limited to primitive manipulations of familiar objects located in habitual surrounds (think of a chimpanzee fitting together sticks or piling up boxes to reach a hanging fruit). Such manipulations are triggered by the current sensory inflow (the reward and the objects are within the field of view) and guided by the sensory-motor feedback in the course of object manipulation. To the extent the process involves self-directed (purposeful) coordination of behaviors, it manifests in rudimentary understanding. 

Note the critical limitations of such rudimentary understanding:

(1) Objects are in the immediate proximity of the animal (within the sensory-motor feedback loop);
(2) Objects have familiar properties and are proximal in space and time, that is, have been co-occurring in the animal’s past history (accordingly, the corresponding packets occupy proximal positions in a small neighborhood in the packet network);
(3) Manipulations are within the envelope of instinctive (genetically determined) responses (e.g., reaching, pulling, dragging, grabbing, etc.).

The “small neighborhood” limitation is particularly significant: on the account of the present theory, transition from protohuman to Sapience was the result of overcoming the limitation. 

#### 3.1.5. Cognitive Revolution: Emergence of Human Understanding

Capital letters in the title indicate that the term refers to the emergence of Sapiens about 80,000 years ago [3]. The hypothesis is that the transition from protohuman to Sapiens consisted in the acquisition of mechanisms allowing:

(1) Coordinating packet vectors across unlimited spans in the packet network; and
(2) Conducting such coordinations without motor-sensory feedback (i.e., while withdrawing from sensory inflows and suppressing overt motor activities).

**Figure 7:**
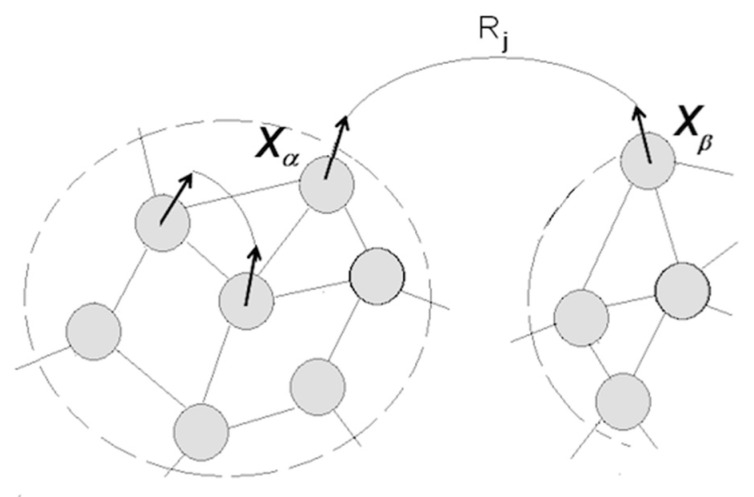
The definitive feature of human intelligence is the ability to construct mental models, by establishing coordination between packets residing in different neighborhoods separated by indefinitely large spans in the associative networks. In this way, relationships can be established between objects and events separated by indefinitely large expanses in time and space (e.g., Newton’s theory allowed people to establish relationships between terrestrial and celestial objects and events).

Stated differently, the transition to Sapiens consisted of the acquisition of understanding capacity, which boils down to self-directed construction and manipulation of mental models comprising packets from distant neighborhoods in the packet network. Figure 7 illustrates this notion. 

Combining coordinated packets into groupings amenable to subsequent inter-group coordination produces a growing hierarchy of relational structures expanding the reaches of understanding. Establishing relationships among packets involves complex neurons responding to different activity patterns in the packets (more on that in Section 5.2.5). Note the broad range of cognitive capacities enabled by the mechanism in Figure 7:

(1) If packet $X_{\alpha }$ corresponds to a currently perceived object A, establishing coordination $(X_{\alpha }$ $R_{j}$ $X_{\beta })$ allows one to attribute causes of A’s behavior to some object B, which is not amenable to perception;
(2) $\mathrm{Coordination}\;(X_{\alpha }$$R_{j}$$X_{\beta })$ suggests the use of object B and deployment of coordination $R_{j\;}$ in order to produce some desired changes in the behavior of object A;
(3) $\mathrm{Coordination}\;(X_{\alpha }$$R_{j}$$X_{\beta })$ allows prediction of changes in B following changes in A;
(4) Exercising coordination $(X_{\alpha }$ $R_{j}$ $X_{\beta })\;\mathrm{amounts}\;\mathrm{to}$ simulating interaction between A and B;
(5) A coordinated pair becomes a functional unit $(X_{\alpha }$ $R_{j}$ $X_{\beta })\;$→ $Y_{k}$ that can be coordinated with other units ($Y_{k}$ $R_{q}$ $Y_{p}$) → $Z_{t}$, and so on;
(6) Establishing coordinations equates to production of information, yielding reduction of entropy, and a growing degree of order in the system. Accordingly, information can be sought that facilitates entropy reduction, via self-directed construction, expansion, and integration of models;
(7) Establishing coordination $\left(X_{\alpha }\;R_{j}\;X_{\beta }\right)$ is experienced as attaining understanding, or grasping the meaning of behavior variations in A and B;
(8) Understanding enables explanation.

A simple example will illustrate these notions. Consider a system of two packets comprising one neuron each and representing “switch” and “light bulb” in a room (switch can be UP and DOWN and bulb can be ON and OFF), and let these packets form in the brain of a person unfamiliar with modern technology. When visiting the room, the person witnesses changes in the state of the bulb. Being perceptive, she also notices changes in the state of the switch but initially fails to make a connection. Understanding emerges when a model is formed comprising both packets and establishing coordination in the behavior of the objects (UP–ON, DOWN–OFF). With that, prediction and explanation become available (e.g., predicting what will happen with the bulb if the switch is turned up or down, explaining what caused the bulb to be on or off). Mental simulation underlies prediction and explanation; attention needs to be shifted between the packets and packet vectors need to be rotated so that coordination in the rotation patterns can be established.

Assume now that the person eventually runs into a situation when turning up the switch fails to turn on the bulb (and continuing flipping the switch did not help). The model would disintegrate into uncoordinated packets, unless a third object (for example, “fuse”) was introduced, with the corresponding neuron forming a separate packet or being absorbed into the packet holding the “switch” neuron. In the latter case, switch and fuse merge in the person’s mind into a composite object (“controller”) whose states are determined by the states of the constituent neurons (UP–ON, DOWN–OFF, UP–OFF, DOWN–ON) and coordinate with the states of the bulb. If familiarization with the modern technology continues, the model for controlling illumination will be combined with other models (e.g., controlling temperature, etc.) subsumed in a model of “room control”, which would be a component in “house control”, and so on, forming a hierarchy amenable to indefinite expansion. At some future point, the person might be able to associate failed illumination with a fallen tree and broken wires observed at some point in the past in a location distant from the house, as in Figure 7. Moreover, Figure 7 implies eventual development of the ability to form models combining concepts acquired at different points in time and belonging to different knowledge domains (neighborhoods) (e.g., the idea of “neuronal packet” amalgamates concepts from neuroscience, thermodynamics, vector algebra, and optimal control theory). 

The term “understanding” denotes “the capacity to apprehend general relations of particulars” (Webster’s Ninth New Collegiate Dictionary). Figure 7 and the above example indicates that understanding advances by grouping variables (the “particulars”) into quasi-stable packets and forming hierarchies of relational structures. Algebra defines relations as mappings between sets (i.e., sets of neurons in packets). Figure 7 expands that notion, defining relations as mappings between sequences of variable combinations (excitation-inhibition patterns) defined in the sets and expressed in the formalism of coordination in the rotation of packet vectors. The contention is that any relation (A controls B, A threatens B, A loves B, etc.) can be expressed in that formalism. 

Note the key differences between learning (registering co-variations) and understanding via constructing models:

(1) Understanding entails entropy reduction, i.e., the resolution of uncertainty or expected surprise. Establishing relations (e.g., switch *controls* bulb) amounts to forming dependencies between packets, which is accompanied by producing information and reducing entropy,
  $$H(X_{\alpha }R_{j}X_{\beta })<H(X_{\alpha },X_{\beta })$$
  here H ($X_{\alpha },X_{\beta }$) denotes entropy before the dependency (relation, coordination) was established.
(2) Understanding entails generalization, i.e., an increase in the marginal likelihood of internal models following a reduction in model complexity. Grasping a relation in a particular process enables transferring it to a variety of other processes different from the original one (e.g., having comprehended that switch *controls* bulb, the person can figure out how to handle desk lamps, floor lamps, fans, or other devices operated by switches, etc.). As formulated by Piaget:
  “…the subject must, in order to understand the process, be able to construct in thought an indefinite series ….and to treat the series he has actually observed as just one sector of that unlimited range of possibilities”.
  [6] (p. 222)
(3) “Understanding brings out reason in things” [6] (p. 222), and thus enables explanations (“the bulb turned on because *this* switch *controls* it and it was *turned up*”).
(4) Most importantly, understanding makes it possible to overcome the inertia of prior learning, and thus enables coping with disruptive changes and unprecedented conditions. Technically, intrinsic to modeling is the possibility of constructing, in thought, various packet groupings until a composition emerges fitting the situation at hand, and thus allowing explanation and prediction.

To summarize, the following suggestions have been made (admittedly, the suggestions are tentative, requiring further development and substantiation):

(1) Intelligence derives from biophysical mechanisms allowing self-directed construction of mental models, establishing coordinated activities in neuronal packets residing in different domains in the packet network. Cognitive functions enabled by the mechanism range from figuring out methods for handling physical objects in order to achieve some desired objectives, to formulating scientific theories defining coordination between abstract variables.
(2) Modeling entails entropy reduction. As a result, the process motivates extracting and producing information that is conducive to further entropy reduction, and thus has intrinsic worth to the system. Accounting for internal information production modifies Equation (1).
  (2) $$H+dI+dI_{\phi }\geq \;0$$
  here $dI_{\phi }$ is the internal information increment. Entropy reduction inside the system—dH is compensated by entropy increases in the surrounds, keeping the overall process in line with the second law.
(3) Along with maximizing intrinsic significance, modeling serves to maximize extrinsic value (utility) by supporting “mental simulation”, thus reducing prediction errors (minimizing variational free energy [11]). To underscore: In feedback-controlled coordinations, information has no intrinsic worth independent of the external conditions it signifies. Decoupling from feedback gives rise to intrinsic worth commensurate with the degree of entropy reduction the information obtains. The pursuit of intrinsic worth involves re-organizing and unifying mental models and seeking information that is subjectively significant, that is, conducive to further entropy reduction. Intrinsic worth motivates cognitive effort in search of understanding.
(4) The overall functional organization of the regulatory system is hierarchical, as shown below. 
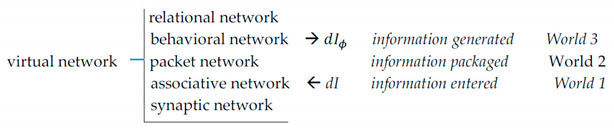
 Synaptic wiring at the bottom gives rise to informational hierarchy, where each upper level is produced by operations on the lower one. The hierarchy extends upward indefinitely (models comprising models comprising models...).
(5) The world is intelligible because its representation is constructed by the same mechanism that is employed in the attempts to understand it. Intelligibility does not equate to ready understanding, it only implies that understanding can be reached eventually with effort.

### 3.2. Supportive Experimental Findings

#### 3.2.1. Neuronal Packets—Are They “Real”?

Returning to Figure 7, imagine energy barriers surrounding packets $X_{\alpha }$, $X_{\beta }\ldots \mathrm{and}$ creating an energy landscape across the network. Energy barriers constitute Markov blankets affording a degree of statistical independence and stability to neuronal groupings inside packets [33]. Establishing coordination $(X_{\alpha }$ $R_{j}$ $X_{\beta })$ requires navigation of the landscape and crossing of the barriers, which incurs energy costs. Rotating packet vectors without crossing the barrier packets presents low energy demands, whereas accessing a packet can be more costly (depending on the height of the barrier), and adjusting packets (re-distributing neurons, as in Figure 2d) is expensive. The system strives to minimize expenditure and maximize returns.

These ideas allow verifiable predictions. For example, it can be expected that packets get sculpted for stability and re-combination: the system strives to obtain a minimal number of packets that can be re-combined to cover broad ranges of condition variations, with the relative packet sizes reflecting variation amplitudes. 

**Figure 8:**
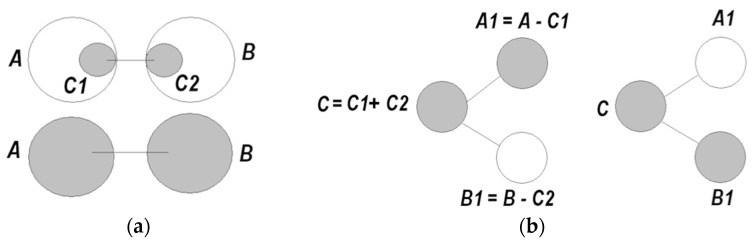
(**a**) Recurring co-activation in groups *C1* and *C2* in assemblies *A* and *B, C1* ⊆A, *C2* ⊆B can “interfacilitate” association between *A* and *B* [34]. (**b**) It was suggested that overlapping activation results in the extraction of the overlap component C and formation of a triad, where the overlap component can coordinate alternatively with the other two members of the triad [30,32].

More precisely, the classical theory by Donald Hebb ascertains that overlapping activity in assemblies *A* and *B* create “interfacilitation” between *A* and *B* (that is, if activity in some subset $C_{1}$ in *A* alternates with activity in subset $C_{2}$ in *B*, *A* and *B* can become associated) [34]. Notwithstanding this possibility, the energy efficiency criteria entails a hypothesis that the overlap component *C* ($C_{1}$ + $C_{2}$) can be separated into a packet that is coordinated alternatively with the adjusted packets, *C* ⇆ (*A* − $C_{1})$ OR *C* ⇆ (*B* − $C_{2})$ (⇆ denotes coordination) [30,32]. Figure 8 explains this important notion. 

With sufficiently sensitive techniques for monitoring neuronal activities, these tendencies in the formation of packets can be detected. Such techniques appeared in the last decade. This section references experimental findings that appear to be consistent with the tendencies predicted in Figure 8, and also provide other evidentiary support for the idea of neuronal packets.

In recent studies [36,37,38,39], state-of-the-art monitoring and analysis techniques applied to a simple animal (medicinal leeches) revealed that an animal’s behavior is regulated by what was termed “shifting neuronal coalitions.” Leeches engage in swimming or crawling, which involves “crawling” assembly A (108 neurons), “swimming assembly” B (6 neurons), and overlapping assembly C (84 neurons). The ganglion circuit for crawling comprising A and C is reconfigured into one for swimming when C is switched to, and coordinates with, B. Strikingly (but consistent with considerations in [30]), a single neuron in C was found to be responsible for determining which “coalition” to form. Crawling is more demanding than swimming (a broader range of condition variations), requiring a larger packet (108 neurons dedicated to crawling and 6 neurons dedicated to swimming). These findings conform closely to the schema depicted in Figure 8b, which is also corroborated by data obtained in the study of rodents [40], as follows.

Mice were exposed to different startling conditions: free-fall inside an elevator (“fall”), a sudden gush of air (“gush”), and earthquake like shaking (“shake”). Large-scale ensemble recordings revealed formation of neuronal “cliques” in the hippocampus that were of the “specialized” and “overlap” types (e.g., “general startle” is an overlap clique responding to any of the three conditions, the “gush or shake’” clique responds to any of the two conditions, “gush” clique responds to one, etc.). Such cliques can be combined economically depending on the subjective significance attached to the situation, e.g., a weak response in the “general startle” clique can be ignored, while a stronger one can suggest attending to the matter). Cliques are “self-organizing, arising out of internal structures and connectivity of neural networks upon behavioral experiences” [40]. 

Critical support for the notion of packet vector rotation notion is provided by seminal studies by Apostolos Georgopoulos and colleagues, identifying neuronal assemblies as the substrate of motor control [41,42,43,44]. Neurons in the motor cortex are directionally selective, the sums of neuron response vectors within assemblies establish assembly vectors that were found to track overt movements exercised by the animal. Movement control (initiating and directing movement) was determined to involve rotation of assembly vectors. It was hypothesized that the vector rotation mechanism is exploited in other forms of cognitive activity [45].

The present theory ascertains that the mechanism of packet vectors is ubiquitous, playing the central role in perceptual and higher-order cognitive processes [30,33]. Recent findings concerning face recognition appear to support this generalization [46]. The fMRI recordings were obtained from high-level sensory neurons in the middle lateral and anterior medial area in macaque brain during face recognition. It was determined that faces are encoded in 50-dimensional (50-D) population vectors, where each neuron’s firing rate was found to be proportional to the projection of a particular incoming face feature onto a single axis in the feature space. As a result, every individual face is encoded as a 50-D vector [46]. 

**Figure 9:**
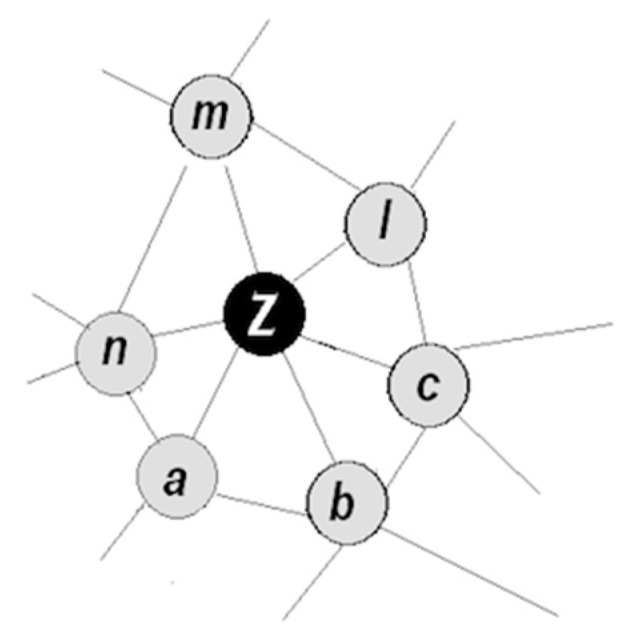
Experiencing energy barriers during recall efforts (adopted from [47]). “Call the forgotten thing *Z*, the first facts with which we felt it was related to *a, b,* and *c*, and the details finally operative in calling it up 1, *m*, and *n*. The activity in *Z* will at first be a mere tension; but as the activities in *a, b,* and *c* little by little irradiate into l, *m*, and *n*… their combined irradiations upon *Z* succeed in helping the tension there to overcome the resistance, and in rousing *Z* to full activity. Through hovering of the attention in the neighborhood of the desired object, the accumulation of associates becomes so great that the combined tensions of their neural processes break through the bar, and the nervous wave pours into the tract, which has so long been awaiting its advent” [47].

Finally, energy barriers can be considered “real” because they manifest in one of the most common and ubiquitous experiences. A vivid account of the experience was given by William James [47], as illustrated in Figure 9.

To summarize, the notion of neuronal packets expands the foundational idea of neuronal assembly [34,35] in a manner consistent with the current theories of neuronal processes underlying cognition [10,11]. The notion is supported by experimental findings and explains naturally some of the most common subjective experiences accompanying cognitive activities. 

#### 3.2.2. Mental Modeling: From Fitting Sticks to Landing on the Moon

This section is central to the article: it purports to explain how the ability to coordinate rotation of packet vectors propelled Sapience, figuratively, from fitting sticks and sharpening stones to landing on the moon and creating intelligent artifacts, all in a period of less than 100,000 years (a blink on the evolutionary time scale). 

Observe that packet vectors establish “bridges”, through which the mental domain is connected to the physical and biophysical domains, as follows. A chimpanzee intending to reach an object causes rotation of the corresponding population vectors in the motor cortex [41,44]. The animal controls the position of the vector but is neither aware of, nor has any control over, the behavior of neurons in the population. The biophysical properties of neuronal substrates are such that rotation of packet vectors obtains coordinated neuronal activities (excitation/inhibition patterns) inside packets consistent with the movement of the vector. Moreover, since multiple muscle groups might be engaged in implementing the intent, coordination is established across multiple packets in the motor cortex, with no or minimal awareness of, and attentive control by, the animal. To reiterate, neurons serve as basic elements in the regulatory system due to their ability to form collectives acting as synergistic wholes, i.e., engage in collective behavior responsive to a macro parameter (position of packet vector) amenable to attentive variation. In that sense, packet vectors connect the mental domain (imagining, volitional control) to the biophysical (inhibition/excitation patterns) and the physical (external objects) domains. 

Think now of a chimpanzee fitting together sticks A and B in order to reach a hanging fruit. The operation suppresses degrees of freedom in A and B and obtains composite C, treated as a functional unit. Accordingly, a mental model is formed, where packets $X_{\alpha }$ and $X_{\beta }$ are combined into composite $Y_{\chi }$. A similar model will be formed by a human engaging in the same task. The crucial difference lies in the way the models are deployed—the animal deploys the model by carrying out the physical manipulation, under the guidance of sensory-motor feedback; by contrast, the human can exercise the model “in the mind”, without the feedback. Think of connected sticks and imagine side A being lifted up. An image “comes up”, where the position of side B is coordinated with that of side A. That is, the human model acquires the property of a synergistic whole amenable to mental manipulation, while animal models have no such property. Stated differently, a human comprehends relations (A *connects to* B) and understands the task, while the animal carries it out without understanding. The significance of this distinction has long been appreciated in cognitive psychology [6], as follows. Young children were presented with a pile of playing cards and asked to construct a “house.” The solution went through multiple attempts involving different forms of selecting and manipulating the cards (e.g., selecting cards based on pictures, holding one card in each hand and setting them up vertically, etc.), until coming to a realization that cards must lean against each other and that coordination (A *leans against* B) imposes reciprocal constraints on the behavior of both components (i.e., if A leans against B, it is not only held by B but also immobilizes B). The attainment of understanding was accompanied by crucial changes in the child’s mental processes: the feedback-directed activity (e.g., handling of the cards) is internalized, allowing subsequent mental activities to be decoupled from the feedback. With that, the “step-by-step material coordinations” are replaced with “co-instantaneous mental coordinations.” More precisely,

“There is, in fact, a very appreciable difference between the two types of co-ordination, the first having a material and causal character because it involves a co-ordination of movements, and the second being implicative. The co-ordinations of actions … must proceed by systematic steps, thus ensuring continual accommodation to the present and the conservation of the past, but impeding inferences as to the future, distant spaces, and possible developments. By contrast, mental co-ordination succeeds in combining all the multifarious data and successive data into an overall, simultaneous picture, which vastly multiplies the powers of spacio-temporal extension, and of deducing possible developments”.

[6] (p. 219)

Implicative, feedback-decoupled coordinations allow construction and manipulation of mental models comprising indefinitely large multitudes of packets, while, crucially, employing a small number of attentive, step-by-step variations in the model. For example, one can think of connecting stick A to stick B, B to C … to Y, and realize that lifting up A will cause position change in Y, without having traced position changes in B, C, ..., X. Obtaining the result (change in Y) in lieu of step-by-step operations implicates a model acting as a synergistic whole; the position of any packet vector constrains positions of all the other packet vectors in the model. To fully appreciate the benefits of such synergy, consider challenges posed by complex coordination tasks, such as playing chess. In particular, it was observed that master players perceive positions

“In large complexes, each of which hangs together as a … functional or dynamic unit. Such a complex, an interrelated knot of pieces … is to be considered as a unit of perception and significance”.

[48]

Accordingly, master-level performance (i.e., guided by the understanding the game)

“consists essentially of taking stock of the spatial, functional, and dynamic relations among the perceived parts, so that they can be combined into one whole”.

[48]

A model combining “complexes” into a unified “whole” obtains “co-instantaneous co-ordination”, eliminating superfluous degrees of freedom in the movement of individual pieces (losing moves don’t come to mind of a master, no more than illegal moves come to the mind of a novice [48]). Stated differently, thinking of moving a particular piece constrains movement of the other pieces, leaving only a few promising moves for further reasoning. Reasoning is a step-by-step process demanding concentrated attention, while the process that constrains reasoning operates automatically (one realizes consequences of moving a chess piece in the same way one realizes consequences of moving a stick connected to other sticks, or moving a card in a “house of cards”, etc.). Stated differently, understanding brings a few moves to the focus of awareness, while placing a multitude of other ones temporarily outside the bounds of awareness. Synergistic models collapse combinatorial complexity inherent in the game, and make reasoning of future moves possible in the context of the entire position and the long-term consequences (e.g., looking ahead 15 moves [49]). Reducing complexity equates to reducing energy demands; modeling works because the need for energy-demanding reasoning is radically reduced by low-cost coordination processes, limiting the amount of reasoning. These ideas are summarized in the following hypothesis: 

Understanding capacity is rooted in the ability of neuronal packets to engage in collective behavior (changes in any packet constrain changes in all the other ones in the model), controlled by parameters amenable to attentive variation. Reducing the number of degrees of freedom in the model eliminates redundant steps in assessing the impact of variations and cuts down energy costs. More technically, following analysis in Stratonovich [20], absorbing information in packet $X_{b}$ about the rotation of the packet vector in $X_{a}$ enables thermal energy to be drawn from the surrounds, and work to be conducted on re-positioning the packet vector in $X_{b}$, consistent with the movement in $X_{a}$. Establishing inter-packet coordination amounts to production of information and reduction of thermodynamic entropy (negentropy generation) in the volume of the model.

Decoupling from the sensory-motor feedback liberates the cognitive system from the directives of the current information inflows, and crucially, from the directives of prior learning. The ability to construct coordinations appropriate for the current intent and conditions is not restricted by the precedents: the system can overcome the inertia of prior learning and form novel constructs, albeit with effort). Novel constructs in performing a task are the product of understanding the task. In that sense, human cognitive activities involved in designing a gadget for reaching a piece of fruit and designing a rocket for reaching the moon are different in scope, but not in substance. Language processing is an integral component of these activities, as discussed in the next section. 

#### 3.2.3. Language

Constructing a model comprising packets $X_{\alpha }$ $\mathrm{and}X_{\beta }$ involves suppressing redundant degrees of freedom in $X_{\alpha }\;\mathrm{and}\;X_{\beta }$ (coordination) and forming a composite unit ($X_{\alpha }$ $X_{\beta }$) → $Y_{\chi }$ amenable to re-combination with other units. This construction process appears to be in close correspondence with a recursive procedure, which according to a recent theory [50], underlies the emergence and operation of language. The procedure (dubbed Merge) operates on symbolic objects (labels, words) and combines pairs of such objects into units amenable to combination with other units ($L_{\alpha }L_{\beta }$) → $L_{\chi }$.

“We can picture Merge’s output as a kind of triangle—the two arguments of Merge form the two legs of the triangle’s “base,” and the label sits on “top” of the triangle”.

[50] (p. 114])

Regarding the present theory, “symbolic objects” [50] (labels) are attached to packets, coordination forms (relations), and coordinated constructs. An important insight supports this contention

“Language evolved as an instrument of internal thought, with externalization as a secondary process”.

[50] (p. 74)

What internal purposes are served by labels? Coordination alternates between accessing the internals of $X_{\alpha }$ (see Figure 9) and exiting $X_{\alpha }$ and shifting attention to $X_{\beta }$ (i.e., alternating between packet vectors and vector components). Arranging multiple packets $X_{\alpha },$ $X_{\beta },\ldots \;X_{\zeta }$ into a coordinated model might require repetitive access, associating high energy cost with the process. Labels are presumed to be implemented as fixed neuronal groupings associated with packets and having minimal sensory contents, sufficient only for making the labels distinct. Different labels designate different contents, which makes fluently shifting attention between packets possible, and in a purposeful manner, while avoiding the expense of entering packets and examining their contents. 

With that view, the emergence of language was the result of decoupling cognitive processes from the sensory-motor feedback, prompted not so much by the need to name external objects but by the need to shift attention fluently between packets representing objects, while combining such packets into mental models. For example, the thinking involved in contemplating lunch and selecting an apple and toast from a menu is carried out on two levels: Merge is applied to labels “apple” and “toast”, and contents of the corresponding packets are accessed and experienced. Symbolic constructs (“apple, toast”) allow fluent attention shifting, preventing attention capture in one of the packets (an ardent apple connoisseur captivated by imagining an apple (sweet, tender, juicy, etc.) can have a hard time diverting attention to review other items in the menu). In short, language processing in both comprehending speech and inner thinking involves alternating between merging symbols and manipulating the packets the symbols designate. In both cases, symbols facilitate model construction by cutting down energy costs. Recent findings [51] appear to support this opinion. 

Levels of neuronal activity were recorded in language-related areas comprising superior temporal and inferior frontal sites during word-by-word sentence presentation. Activity increased with each successive word in a sentence, but decreased abruptly whenever words could be merged into a phrase. It was hypothesized that such abrupt decreases accompany formation of a new neuronal population vector, orthogonal to those of the merge constituents [51]. On the present view, activity increases indicate seeking coordinations (“fitting” the packets together, not unlike fitting sticks together), followed by abrupt decreases when coordinations (relations) are established. These suggestions appear to agree with the “symbol grounding” theory of meaning—apprehending meaning in a phrase involves indexing words to objects or perceptual analogs, defining affordances from the objects and analogs, and meshing the affordances under the guidance of the syntax [52]. 

Since language processing involves construction of models, language comprehension is predicated on the availability of the requisite coordinations. Accordingly, limited availability entails a comprehension deficit. For example, infants reach out for a toy placed under cover $X_{1}$, but when, in their full view, the toy is removed and placed under a different cover $X_{2}$, they keep reaching under $X_{1}$ [53]. An adult with a similar deficit cannot fully comprehend phrases like “I put an apple in a basket and then moved it to another basket”, even if the ability to form grammatical sentences is intact and all the words are understood. 

It can be hypothesized that the transition from protohuman to Sapience was prompted by a confluence of two developments: (a) synergistic mechanisms of sensory-motor coordination optimized for the manipulation of external objects [54,55,56,57,58,59] were co-opted and re-purposed for the manipulation of mental objects, and (b) signaling mechanisms were re-purposed for internal labeling (perhaps, transition from signaling-to-others to “signaling-to-oneself” was preceded by the ability to suppress or delay signaling, e.g., to avoid attracting a predator’s attention). 

To summarize, language facilitates construction of models by enabling manipulation of packets without accessing their contents. Comprehending language involves a process that alternates between syntactic objects and the corresponding packets. In essence, language continues the trend of decoupling mental processes from sensory inflows: decoupling from the motor-sensory feedback is followed by decoupling from the packets’ sensory contents. The benefit lies in the enhanced ability to juxtapose packets from different neighborhoods (see Figure 7), “which vastly multiplies the powers of spacio-temporal extension, and of deducing possible developments” [6] (p. 219).

## 4. Aspects of Human Cognition

Part 3 focused on information dynamics underlying understanding. This part discusses other aspects of human cognition overlapping with that of understanding. 

### 4.1. Landscape Navigation in Norm and Pathology

Access to memories can be lost as a result of erosion in the synaptic network, or hampered by abnormal conditions in the landscape. Working conditions require easy access (see Figure 8) and easy exit. Stated differently, the barriers must be high enough to allow sustained attention concentration within packets and low enough to prevent attention capture. The height of energy barriers *F* is determined by the amount of free energy in the phase surface (Markov blanket [10]) separating packet internals from the surrounds, *F* = *U* − *TH*, here *U* is intra-packet energy and *H* is packet entropy, and *T* is temperature. Setting aside methods for calculating the values *U* and *H*, this section considers regulation of energy barriers by varying temperature, under the assumption that temperature correlates with arousal and activation (local temperature during brain activation is determined by the balance between heat evacuation in the blood flow and metabolic heat generation [60]. Activation and arousal are distinct, but probably interrelated, aspects of brain energetics).

**Figure 10:**
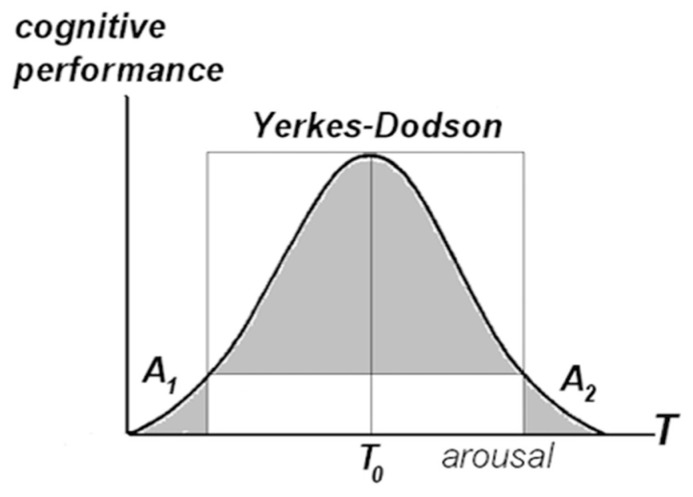
Temperature-regulated access to packets explains near-optimal performance within the Yerkes–Dodson range and degraded performance at the temperature extremes ($A_{1}$—autism spectrum pathology, $A_{2}$—Alzheimer’s spectrum pathology). $T_{0}$ denotes optimal temperature.

At the first approximation, the “temperature–barrier height” relationship is straightforward: the higher the temperature, the lower the barriers. Accordingly, it can be suggested that the Yerkes-Dodson law of cognitive performance [61] captures the dependency between the level of arousal and cognitive performance within a “medium” temperature range. At the boundaries of the range, high arousal hampers concentration, while low arousal (stress, depression) hampers attention-shifting necessary for accounting for, and integrating different aspects of, the task. Figure 10 suggests that temperature deregulation entails Alzheimer’s type (high temperature) or autistic type (low temperature) performance pathology. In the former case, packets dissolve, turning objects into shapeless “blobs”, and eventually transforming the world into undifferentiated flux. In the latter case, the network is fragmented into isolated “islands”, with no traffic between them. 

Figure 10 defines changes in cognitive performance in norm and pathology as a continuum over a single variable. The simplifying proposal in Figure 10 is not inconsistent with the data, as follows.

In general, people with autism spectrum disorders present abnormalities in the connectivity of brain systems. Studies of cortical activation and cerebral metabolic landscapes in autism consistently indicate low correlation between cortical areas, including functionally impaired interactions between frontal and parietal, neostriatum, and thalamic areas involved in directed attention [62,63,64]. Autistic performance is often accompanied by depression [65], in continuation of a similar condition in the low-temperature tail within the Yerkes-Dodson range. The notions of fragmented packet network and attention confinement in isolated fragments are consistent with the savant syndrome, where “islands of genius” stand in sharp contrast to the overall cognitive handicap [66]. 

Cognitive degradation in Alzheimer’s disease is considered to be caused by deposition of plagues consisting of amyloid peptides (imbalance between peptide production and clearance is presumed to trigger a cascade of degenerative developments leading to synaptic injury, and eventually, demise [67]). It can be speculated that deposits constrict blood flow, thus hampering heat removal. Conceivably, other elements in the cascade produce degenerative changes that summarily entail dissolution of packets, while the synapses are still intact. In that scenario, the onset of performance degradation can take place before the system gets overwhelmed by synaptic erosion and loss (i.e., per Figure 9, one can no longer recollect Z or recognize Z because Z has merged into the surrounds, although the synapses have not been disrupted. For the same reason, new packets cannot be formed, i.e., sensory elements will not integrate into “objects”). 

Some symptoms of vascular aphasia (produced by lesions) can also be produced by de-regulation in the energy landscape (logorrhea, or nonstop speech, adynamia, or absence of attempts to speak, perseveration, inability to name objects, inability to carry out simple instructions if they require shifting attention between objects, etc.). More generally, disintegration of neuronal networks in different pathological processes appears to be an emergent theoretical framework providing a unifying account of heterogeneous brain disorders [68]. The notions of pathological distortions and de-regulation in the energy landscape appear to be consistent with that framework. 

### 4.2. Cognitive Effort, Value Attribution, and Consciousness

Moving through energy landscape and manipulating packets expend energy, and thus require effort. The experience of exerting and focusing such effort is co-extensive with the experience of consciousness. Arguably, Descartes’ “cogito ergo sum” expresses a similar idea: In view of Descartes’ insistence on building philosophy on the foundation of the most basic facts impervious to doubt [69], the “cogito ergo sum” thesis seems to be pointing at the effort of thinking as the most direct and unquestionably certain experience that is co-extensive with the experience of Self. To underscore, it is not the content of thinking but the effort that Self keeps investing in the thought process that constitutes the only direct and fundamental experience of which Self can have no doubts about, because by initiating the effort, and while continuing it, Self becomes and continues to be aware of Self as the source of the effort.

More precisely, it was demonstrated that conscious awareness of own intentions (e.g., intending to move) is preceded by subconscious activities in frontal, parietal, and other brain areas [70]. These findings inspired suggestions that consciousness is an epiphenomenon, that is, a post-factum process that creates an illusion of self-control without playing any role in the actual mechanisms responsible for behavior organization. The present theory offers a different view, as follows. As stipulated in Part 1, thinking involves priming packets followed by suppressing degrees of freedom and coordinating activities across packet compositions. The initial stage of packet priming can remain below the threshold of conscious awareness, while efforts required for constraining intra-packet variations (“fitting” the packets together in a synergistic whole) elevate these activities above the threshold. That is, cognitive effort is co-extensive with conscious execution of mental operations. In particular, rotating packet vectors can be conceptualized as regulatory activity requiring effort *η*(*τ*) commensurate with the time constraints and changes in the vector’s angular position φ(t):

(3) $$\eta t=\frac{d^{2}\varphi \left(t\right)}{dt^{2}}+\frac{d\varphi \left(t\right)}{dt}$$

More generally, effort *η* is a function of changes in the rotation trajectory (e.g., figuring out how an object habitually used for purpose 1 (trajectory 1) can be used in some novel way in the service of purpose 2 (trajectory 2)). Depending on the distance between the trajectories (resistance to change measured in appropriate units) and subjective limitations, sufficient effort might not become available within the required time period, or ever (the idea never comes to mind). 

During sleep, the regulatory function is disengaged, *η* = 0, entailing the characteristic experience of facing hallucination-like conditions that one can neither change nor escape from (more on that in the next section). In pathology (e.g., schizophrenia), degradation of the regulatory function manifests in the experience of “thought insertion”—patients report having thoughts controlled by someone else. Such patients can usually maintain distinction between the thoughts they own and control (*η* > 0), and those they attribute to an alien source beyond their control [71]. 

Mobilizing and sustaining cognitive effort is predicated on motivation, and thus involves selecting and concentrating on highly significant targets, keeping the magnitude of the effort commensurate with the significance level attributed to the target. Accordingly, prioritization is an integral component of consciousness. Decision making is a balancing process weighing the expected amount of effort against the expected gain [72,73]. The overall tendency towards maximizing gain while minimizing efforts does not exclude periods of sustained concentration and elevated efforts, consistent with the requirements of long-term performance optimization (a net gain maximization over long time periods). In perception (i.e., vision), target prioritization influences velocity and duration of saccadic movements (more significant targets attract faster saccades) [74]. Prioritization influences predominantly long-latency saccades (short-latency saccades are determined mainly by salience) [75]. In thinking, cognitive tasks are accorded different relative significance determining the magnitude and duration of the cognitive effort. In pathology (e.g., obsessive-compulsive disorder), abnormally prioritized (over-valued) ideas are associated with a high degree of affect and can preoccupy the patient’s mental life [76]. 

The critical role of significance attribution in the organization of cognitive effort is reflected in the richness and complexity of the underlying neuronal substrate. The “valuation system” in the brain comprises the dorsomedial prefrontal cortex, dorsal and posterior striatum, thalamus, and other regions [77]. Reward anticipation, assessment of reward magnitude, and reward consumption appear to engage different (albeit overlapping) subsystems [78,79]. Transformation from objective to subjective significance (subjective utility) is carried out by the dorsal anterior midcingulate cortex (daMCC). Connections between daMCC and other brain regions might provide channels through which contextual information is integrated into utility and significance attribution [80]. 

### 4.3. Assimilation and Accommodation

Terms “assimilation” and “accommodation” were introduced by Piaget [7], denoting, roughly, entering new information (assimilating, or absorbing information) into memory structures and adjusting (accommodating) the previously formed structures to the new entries. The present theory identifies assimilation with the modification of synaptic weights, and splits accommodation into spontaneous changes and those resulting from deliberate thinking. Spontaneous changes involve modification of neuronal packets (shrinking, expansion, dissolution, shuffling neurons between packets) that are presumed to occur during sleep, and, in the awake state, as a result of processes in the “default mode network” [81]. Segregation of neurons by the packet mechanism is balanced by the sleep and default network mechanisms serving to “smooth out” the results of segregation, by “shaking the packets together.” More precisely, increases of temperature across packet networks temporarily bring down energy barriers and enable re-distribution of neurons under global optimization criteria (ultimately minimizing the number of packets while maximizing the number of successful combinations). In the process, smaller and weaker packets can dissolve, with the released neurons getting absorbed into stronger packets, or alternatively, the larger packets can break up [30]. In that way, “shaking together” is a counterpart of segregation, integral to the optimization and regulation mechanisms (according to Koestler, the term *cognition* derives from the Latin *cogitare*, meaning *shaking together* [82]. Crucially, “shaking together” requires both decoupling from sensory inputs and disengagement from attentive regulation. The following findings appear to be in line with these suggestions.

A network (“default mode network”) has been discovered that comprises areas in the medial and lateral parietal, medial prefrontal, and medial and lateral temporal cortices, and sharply decreases its activity in the course of attention-demanding tasks and increases activity during repose. It was shown that such networks exist not only in the human brain but also in those of primates, cats, and rodents, indicating their fundamental role in brain functioning [81]. On the present proposal, the same role, or a closely related one, is carried out by the mechanisms activated during sleep. 

In particular, REM sleep is accompanied by elevated activation in limbic and paralimbic brain regions involved in arousal regulation [83]. Release of arousal-regulating acetylcholine is increased during REM sleep and decreased in the NREM phase [84]. A recently discovered peptide orexin (Orx) is actively discharged during waking and discontinues during sleep. Firing of Orx-discharging neurons is coordinated with the activity of other neurons, so that discharging Orx entails increase of muscle tone during waking and muscle atonia during sleep [85]. Disengaged attentive control and increased temperature “shake up” the system. Arousal and temperature are intertwined—observations of heat-seeking behavior in rats subjected to sleep deprivation [86] seem to provide a degree of support to this notion. 

Some proposals associate memory consolidation during sleep with synaptic changes. The proposal seems to be contradicted by the fact that most dreams fade without trace upon wakening. On the other hand, correlation between elevated pre-learning cortisol levels and memory consolidation during sleep seems to point at *shaking together* as the mechanism of improvement (no synaptic modifications), consistent with the notion that “tagging” of important stimuli by cortisol allows “optimal consolidation of salient information in a selective manner” [87].

It has been suggested that REM sleep constitutes a “protoconscious state” supplying virtual models of the world [88]. REM sleep involves random changes across packets that result from temperature modulation and cause the characteristic experience of hallucinatory images morphing into each other in a kaleidoscopic fashion. Dream interpretation techniques advanced in psychoanalysis (on the notion that “suppressed memories” of traumatic events influence dream contents) appear to be not inconsistent with this proposal, except that “fenced off” could be a more descriptive term than “suppressed”, indicating blockages in accessing memory contents that are lifted during sleep. The often cited occasions of obtaining problem solutions in dreams are exceedingly sparse (like winning a lottery), and usually follow periods of intense concentration on the problem (in the manner depicted in Figure 9). By the same token, coherent dreams are probably much less frequent than reports about them, due to inadvertent embellishment and added coherence when trying to fit the retained material into a narrative. By contrast, the feeling of having a “clear mind” after a good sleep is quite common. In short, sleep facilitates construction of actionable mental models in the awake state but does not supply them. 

### 4.4. Architecture for Coordination 

It appears that inter-packet coordination can involve cortico-cortical connections, and more significantly, cortico-thalamo-cortical pathways running through the higher-order thalamus, comprising intralaminar and medial nuclei. Recent findings indicate that thalamic neurons are responsible for modulating the degree of synchrony between different groups of cortical neurons according to behavioral demands [89,90,91]. More precisely, the anterior group of intralaminar thalamic nuclei receives subcortical input from the cerebellum, brainstem, and spinal cord, and projects to the frontal and parietal cortex. The central lateral and paracentral nuclei project to the lateral cortical areas, whereas the central medial nucleus projects to the medial and basal cortical areas. Pulvinar is reciprocally connected with much of the cerebral cortex: simultaneous recordings from the pulvinar and cortex have shown synchronized activity in these areas during attention tasks. Lesions of the pulvinar have been shown to reduce cortical excitability and produce deficits in attention and sensory-guided actions [92]. Control of movement has been shown to involve the motor thalamus and movement-related central thalamus connected to the cerebellum [93]. Modifying learned behavior under novel conditions (counteracting pre-action bias) was found to involve the centro-median thalamic nucleus [94]. Thalamic structures participate in language mechanisms [95]. In short, the higher-order thalamus appears to orchestrate coordination between cortical ensembles [96]. 

Accordingly, the overall architecture for packet manipulation (rotating packet vectors) and inter-packet coordination in the human brain might involve the higher-order motor and centro-median thalamus, cerebellum [97], basal ganglia [98], and hippocampus [99]. 

### 4.5. From Self-Organization to Self-Realization

Neuronal wiring is genetically determined. Wiring blueprints specifying neuronal types and connections get implemented during embryonic and postnatal development through a series of precisely orchestrated developmental events regulated by specific molecular mechanisms [100]. In turn, inter-cell connectivity and individuality of the interacting cells determine brain dynamics and collective modes of neuronal activity.

It has been hypothesized that the brain (neural system) operates in the proximity of saddle points [101] organized into chains in a high-dimensional neuronal-phase space. More precisely, brain activity unfolds within Stable Heteroclinic Channels (SCH), comprising saddle points, bundles of trajectories condensed in the vicinity of the saddle chain, and unstable separatrics [102]. Extending these ideas, it can be suggested that genetics populates neuronal phase space with virtual attractors expected to be visited during their life time. The virtual attractor chain unfolds in the course of maturation, with transitions between the attractors being propelled by changes in the functional connectivity, i.e., the mosaic of synaptic weights modified by organism-environment interactions. Approaching saddle points causes contraction of phase volume (e.g., in a dynamic system of the type $\dot{x}$ = X(**x**), div X(**x**) ≤δ < 0, phase volume $V_{0}$ during time *τ* contracts to *V* = $V_{0}$ exp (δ τ)). The system seeks environmental inputs (conditions) facilitating its movement along the unfolding attractor chain (hence, the self-realization). This conjecture has two implications:

(1) Genetics determines a person’s intellectual pursuits in the course of the life time and the ability to realize such pursuits within some range of condition variations;
(2) Absence of the requisite conditions can arrest self-realization and cause frustration.

Accordingly, it can be hypothesized that minimizing frustration is one of the organizing criteria in human cognition, concomitant with the surprise minimization principle [11]. The degree of frustration can be related to the extent and duration of deviations from the genetically favored developmental trajectories. 

## 5. Summary and Discussion

This part is broken into three sections. Section 5.1 discusses ideas central to the theory. Section 5.2 clarifies and extends some of the notions in the article, focusing on their interpretations within the framework of the theory (for convenience, this section re-states some of the points scattered throughout the text). Section 5.3 presents a thumbnail digest, emphasizing distinctions between this proposal and other ideas in the literature. Suggestions for further research conclude the paper. 

### 5.1. Discussion: How Neurons Make Us Smart

Challenges facing cognitive systems in a fluid environment can be defined as follows. The present condition in the environment is *A*, changing it to $A_{1}$ promises reward W; which coordinations can I deploy to achieve *A* → $A_{1}$ with an acceptable level of effort and within the available time (i.e., before the opportunity *A* is gone)? In the parlance of neuronal processes, the problem maps onto the following: “Neurons $X_{i}$, …, $X_{n}$ have fired, indicating presence of stimuli $C_{p},\ldots$, $C_{q}$ offering potential energy reward W; therefore, which neurons should be fired next in order to get the reward with sufficient certainty and at the lowest energy cost?”. Operation of the cognitive system is reduced to dynamic optimization of neuronal resources. The remainder of this section applies the mapping to elaborate some of the key ideas in this paper. 

Assume that optimal allocations have been computed for a large set of stimuli. For argument’s sake, assume that all the possible allocations have been calculated meticulously, step-by-step, and the best reward-maximizing, expense-minimizing allocation has been selected, allocating to each stimuli a group of neurons. Record these groups and do the following: heat the neuronal pool and witness formation of neuronal packets, similar to Bernard cells. When comparing packets to the computed groups, you will find that they are nearly-identical. The point is that thermodynamically-driven self-organization produces neuronal packets yielding near-optimal allocations. At the psychological level, the process manifests in the transformation of stimuli streams into sets of distinct objects preserving their self-identity within some ranges of condition variation. The central claim is that near-optimal distribution of neurons between packets is neither computed by hidden agents, nor results from message passing obtaining a negotiated consensus in the neuronal pool. Self-organization is the key property of the neuronal substrate, making it a suitable medium for behavior regulation. Humans are smart, not because their brains run efficient step-by-step procedures, but precisely because neurons engage in collective behavior alleviating the need for such procedures. The emergence of Sapience was a result of a confluence of developments in the nervous system, enabling advanced forms of collective behavior yielding understanding. The capacity is inherent in the species and is mastered by individuals in the course of maturation. 

Thermodynamics works for leeches the same way it works for humans. Leeches possess a model of the world comprised of two object types: crawlable object and swimmable object. The crawlable object behaves in many different ways, all accounted for by different activity patterns in the “crawl” packet (the same goes for the “swim” packet). Swimming and crawling are different activities but have something in common (an overlap). Thermodynamics enforces economy in the form of “shifting coalitions,” by combining the “overlap” packet alternatively with the “swim” or “crawl” packets [38]. Leeches deploy their models in no other way but by crawling and swimming, in a move-by-move fashion. Crawling from point 1 to point 2 and then swimming from point 2 to point 3 is accompanied by packet vectors oscillating around the 1-2 and 2-3 axes (e.g., responding to changes in the crawl surface or conditions in the swim volume). Transforming a leech into a “thinking leech” would require an ability to form models of 1-2-3 movement that can be exercised without performing the movement, and crucially, will orient packet vectors along the 1-2 and 2-3 axes without the need for reproducing the movement-by-movement oscillations. A “thinking leech” will turn into an “understanding leech” when a model can be formed such that thinking “I would rather crawl to point 4 and swim from there” will automatically orient the swim vector along the 4-3 axis. A crawling leech is unaware of the forthcoming swimming while the understanding leech is, and is also aware that changes in crawling will have consequences for the forthcoming swimming. 

In a similar fashion, synergistic models of chess positions allow one to conjure up strategic ideas without thinking through all the moves, with the ideas (if coherent) radically reducing the number of moves that remain to be thought through. The result is that chess machines had to reach the speed of searching about $10^{8}$ moves per second in order to compete with humans capable of considering at most a few moves per minute (Deep Thought (1989) searched about $10^{6}$ positions per second, Deep Blue (1996) searched $10^{11}\;\mathrm{positions}\;\mathrm{per}\;\mathrm{move}$ [103]). The comparison suggests a new interpretation of the “Achilles can’t keep up with a tortoise” paradox—the computing Achilles takes detours running multiple times to the moon and back for every step taken by the understanding tortoise (2000 steps per mile, 240K miles to the moon). No wonder Achilles is energy hungry. 

These suggestions contradict the mainstream cognitive science, where intelligence is equated to possession of algorithms and the role of understanding is downplayed. In particular, a treatise on human problem solving that is foundational in the discipline [103] allowed the issue of understanding to enter the argument once (on page 822), and only to question the role of understanding in performance:

“Observe that a high level of mechanization can be achieved in executing the algorithm, without any evidence of understanding, and a high level of understanding can be achieved at a stage where the algorithm still has to be followed from an externally stored recipe”.

[104]

The argument is not without problems [32], but proved to be compelling enough to cause associating intelligence with acquiring algorithms (learning), while marginalizing the role of understanding. As suggested earlier, understanding exploits the machinery of sensory-motor coordination but decouples the cognitive process from sensory inputs, thus liberating it from the dictates of prior learning. As a result, the responsibility for performance efficiency is shifted from accumulating and searching through precedents to constructing coherent explanations, as in abductive inference:

“Abduction … is an inferential step … including preference for any one hypothesis over others which would equally explain the facts, so long as this preference is not based upon any previous knowledge bearing upon the truth of the hypothesis, nor on any testing of any of the hypotheses, after having admitted them on probation”.

[105]

Explanations enable reliable predictions. For example, one can observe movement of object A, accumulate statistics, and make predictions about future movements. Alternatively, apprehending that (A *is inside* B) will explain peculiarities in the movement of A and predict that whatever the future trajectories, they will not cross the perimeter of B. More generally, understanding involves recursive application of set operation (e.g., alternating between packet vector and vector components) having no algorithmic expression. For example, thinking “my class of A, B, …, Z” can conjure up either a set of images, or a featureless unit, as in “I am transferring my class to another room.” Without having condensed the multitude into a unit, thinking of the transfer would require either its execution, step-by-step and person-by-person (A, and B, …, and Z), or the envisioning of such an execution. If operations on sets were restricted to operations on members, ideas concerning sets as wholes could be neither formed nor comprehended. Hence, no human thinking.

**Figure 11:**
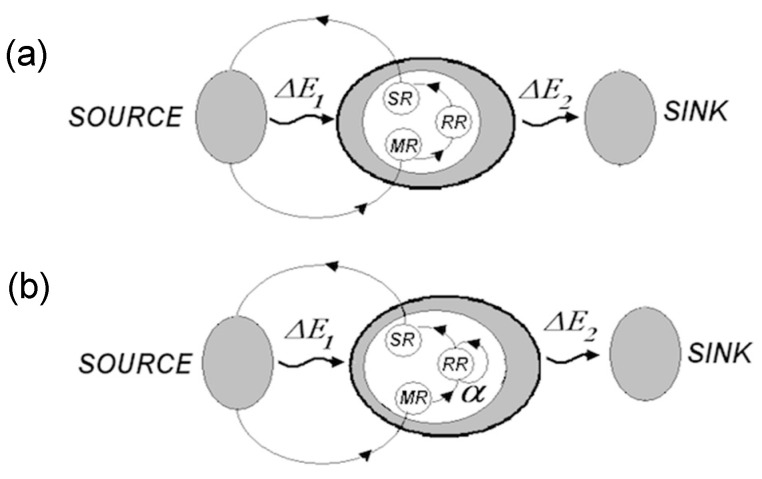
(**a**) The Central Nervous System (CNS) is a regulatory organ comprising neuronal resources of three kinds: sensory resources (SR), motor resources (MR), and regulatory resources (RR), orchestrating deployment of the other two types (sensory-motor coordination). Overt sensory-motor activities change the state of the energy source, seeking to maximize energy inflow $\Delta E_{1}$ → *max*. (**b**) The human CNS includes an extra regulatory loop (α loop), allowing manipulation of regulatory resources and engagement of sensory-motor resources in a manner decoupled from the overt sensory-motor activities. The extra loop is energy demanding but steeply increases regulatory efficiency by maximizing intakes and minimizing losses, $\Delta E_{2}$ → *min.* The understanding capacity derives from the operation of the loop.

Attributing intelligence to the properties of biological neurons does not rule out the possibility of designing intelligent artifacts. On the contrary, apprehending the underlying principles can inform the design of computational methods that approximate biological mechanisms and construction of devices that emulate them. In a similar fashion, apprehending the principles of aerodynamics allowed design of flying machines that do not flap wings or land on trees. Figure 11 summarizes the proposed theory, conceptualizing cognitive processes as allocations of neuronal resources. 

Conceptualizing cognition as dynamic optimization of neuronal resources translates naturally into a computational framework (dubbed “gnostron”), where neuronal resources are allocated probabilistically to streams of reward-carrying stimuli [30,106,107]. The key elements of the present theory (formation of associative networks, formation of packets, packet manipulation and co-ordination, etc.) map directly onto the optimization procedure, with a straightforward interpretation—they represent collective behavior in the neuronal system and serve as heuristics reducing complexity of the procedures with minimal sacrifices of accuracy.

The gnostron framework is orthogonal to that of perceptron (neural network) (dynamically selected neurons versus a fixed set of neurons, feedback-driven operations versus feedback-decoupled operation, other). Increasing internal order in the gnostron system equates to negentropy generation. Boundary energy barriers in packets implement Markov blankets [10]. Optimization of neuronal resources yields surprise minimization, reconciling the principle of variational free energy minimization [11] with the thermodynamically-motivated requirement to minimize energy expenditure and divert free energy to the work of mental modeling [31,33].

The theory offers some predictions concerning the properties of biological neurons and characteristics of neuronal space. In particular, the theory anticipates the existence of hyper-complex neurons (probably in the higher-order thalamus) that respond to different activity patterns in neuronal packets, and importantly, different rates of activity variation [108]. Such hyper-complex and complex neurons can form tensor structures yielding activity patterns invariant under coordinate transformations. The thalamus and cerebellum [109] can operate in a coordinated fashion in the vector space defined by packet vectors. 

It can be expected that the next generation of AI systems will differ from the present one, just as the first airplane by the Wright brothers differs from Boeing 757. The advancement is predicated on elucidating biophysical mechanisms responsible for turning neuronal collectives into synergistic wholes amenable to mental manipulation. The future systems will not be programmed but rather endowed with “genetic” propensities compelling them to develop of understanding of their environment sufficient for fulfilling the operator-defined goals. Insights concerning the design of such systems might come from the analysis of biophysical processes in individual cells [110,111,112], relations between information and energy [113], information dynamics in physiological structures [114,115], or other areas, contributing into the development of an expanded theoretical framework unifying information-theoretic [10,11], physics-motivated [116,117], and biophysical accounts of cognition [118]. Progress towards such unification will enable transition from machine learning to machine understanding.

### 5.2. Clarifications and Definitions

#### 5.2.1. The Brain Operates as a Resource Allocation System with Self-Adaptive Capabilities

This theory conceptualizes the brain operation as a probabilistic resource allocation system with self-adaptive capabilities; neurons are resources dynamically allocated to streams of stimuli [119]. Allocations (accessing, mobilizing, and firing neurons) consume energy, successful allocations are rewarded by energy deposits emitted by stimuli, and self-organization in the system seeks to maintain net energy inflows above the survival threshold [31]. Central to this concept is the notion of self-adaptation, as envisioned by Roger Sperry: “*a brain process must be able to detect and to react to the pattern properties of its own excitation*” [120].

Self-adaptation in the brain entails optimization of neuronal resources under a dual criteria: maximizing energy inflows from the outside, while minimizing energy expenditures in the inside. Attention, motivation, and other functions are defined within this optimization framework. Self-reflective thinking, self-awareness, and self-consciousness are attributes of self-adaptation. 

#### 5.2.2. Attention

On the account of this theory, attention is a brain process that not only reacts to “the pattern properties” in the brain but actively orchestrates them (mobilizes and selectively excites or inhibits neurons). The theory differentiates attention mechanisms operating on external stimuli and those operating on the internal patterns. This view appears to be supported by a number of findings and recent theoretical suggestions, as follows. It is now thought that attention is not a unitary process but involves two distinct neuronal systems. The ventral network implements exogenous (stimulus-driven) attention, while the dorsal parieto-frontal network [121] and anterior insula network implement endogenous (self-directed, volitional, goal-directed) control. The systems converge in the lateral prefrontal area [122]. Coherent behavior is a product of coherent neuronal firings in diverse areas orchestrated by the attention mechanism implemented in corticiothalamic loops [96]. Associating the function of attention with orchestration of firing and inhibition in neuronal networks (see Figure 7) is consistent with the above findings and proposal in a previous study [123]:

“An attentional mechanism helps sets of the relevant neurons to fire in a coherent semi-oscillatory way … so that a temporary global unity is imposed on neurons in many different parts of the brain”.

[123]

Attention alternating between packets in the formation of mental models obtains such global unity. 

#### 5.2.3. Motivation

The concept of motivation subsumes the totality of goal-related processes. Neuronal substrates of motivation include extended amygdala, the ventral striatopallidum, and other subsystems in the basal forebrain [124]. Recently, neurons were identified in the striatum that are sensitive to the motivational context in which the activity is being carried out [125]. Seeking significant information and pursuing understanding constitute goals that conceivably can engage the same neuronal substrate as other goal-related processes. 

#### 5.2.4. Understanding in Humans and Animals

Large amounts of data has been accumulated in the animal studies demonstrating remarkable cognitive capabilities in other species (e.g., numerical capabilities in honeybees [126]) and suggesting that the functions of human intelligence could have evolved from neural substrates common to many species [127]. Recognizing that a significant overlap exists in the principles governing neuronal mechanisms across a spectrum of species [128], this article is focused on cognitive functions that are subsumed in human understanding, and arguably, lie outside the overlap area (e.g., abduction, explanation). The depth of the available functional hierarchy could be one of major differences between humans and animals (suggested by a reviewer). At the present time, the demarcation line between human intelligence and that of other species has not been clearly defined, and is likely to be revised as new data becomes available. 

#### 5.2.5. Neuronal Substrate of Relations

This theory assumes that the emergence of mental modeling in humans involved co-opting mechanisms of sensory-motor coordination optimized for the manipulation of external objects and re-purposing them for the manipulation of internal, or mental objects. On that assumption, establishing relations between objects involves complex and hyper-complex regulatory neurons responding to kinematic variables; that is, not only to packet composition but also to different forms of coordination in the movement of packet vectors (hypercomplex neurons respond to coordination between packets comprising complex neurons). Structures comprising complex and hypercomplex regulatory neurons can implement relations of any complexity [108]. A number of findings appear to suggest the feasibility of the notion, as follows. A recent study identified and modeled neurons sensitive to the instantaneous position, velocity, and acceleration of the stimuli, as well as to short strips of stimulus trajectory [129]. Earlier studies identified directionally-selective neurons responding to movement of the stimulus in the preferred direction [130]. Neurons in the motor cortex have been identified as responsible for the coordinated action of large muscle groups (“muscle synergies”), enabling organized movements of limbs to particular points in space [131,132]. Such complex neurons can be grouped, producing a “vocabulary of neural primitives.” Simulations have demonstrated the feasibility of orchestrating coordinated motor activities by deploying various combinations of such primitives [133]. In general, limits of sensitivity and functional diversity of complex neurons are yet to be determined. For example, a neuron was discovered in the human hippocampus selectively responding to different images of the same person, even if wearing a disguise. Moreover, the same neuron responded to the name of that person expressed in different modalities (written and spoken) [134]. 

#### 5.2.6. Relations as Objects

A particular form of abstract thought (identified by Charles Pierce and called *hypostatic abstraction* [135]) transforms relations into objects. For example, “relation A *loves* B” implies a certain form of coordination in the behavior of A and B. Hypostatic abstraction postulates a universal source of such coordination, treating it as an object separate from A and B (say, a goddess of love) and capable of granting or withholding love (moreover, activities attributed to the source can be further abstracted and treated as objects, i.e., Cupid’s arrows). Both ordinary and scientific thinking involve objectification of properties and relations (e.g., the idea of phlogiston). 

#### 5.2.7. Thinking

Thinking involves grouping, grasping, and simulating—packets are grouped into models, relations between packets are grasped, and manipulating packet contents (rotating packet vectors) constitutes simulation. Insight (in-sight) involves “looking inside” packets, i.e., completing a transition, which requires effort, from being vaguely aware of the packet internals to experiencing and manipulating these internals (please re-visit Figure 9). Grasp is a form of insight resulting in apprehending coordination between patterns of changes in the packet internals. Insight is a routine component of thinking. Reasoning (symbol manipulation) is auxiliary to mental modeling and is enabled by it. An example will illustrate these suggestions. 

Consider a variation of Wechsler’s intelligence test. A subject is presented with a picture showing fragments of a vase lying on the floor next to a vase stand and a cat sitting nearby, and asked to explain the scene in as many ways as might come to mind. Assume three answers: (A) the cat jumped and kicked down the vase, (B) a child was playing with a ball in that room sometimes ago, and (C) a poltergeist did it. With the present theory, these answers were enabled by operations on a mental model comprising three packets (vase, cat, vase stand), as follows. Answer (A) involved grasping a relation (cat *pushed* vase) and imagining the cat jumping and the vase falling from the stand (insight, simulation). Answer (B) involved abduction (outside packets (child, ball) were pulled into the model having no sensory counterparts in the picture; the subject neither had prior knowledge bearing on the hypothesis nor possessed any means for validating it). Answer (C) invoked hypostatic abstraction (transforming relation *push* into object *mischievous pusher*). Note two critical features of mental modeling: (1) Models reduce the number of degrees of freedom in the packets (assume that packet *cat* affords five instantiations: *sitting cat*, *walking cat, running cat*, *sleeping cat*, and *jumping cat.* Of those, only the last option was available. It is safe to assume that images of a sitting or sleeping cat floating through the air were not rejected upon examination but simply did not come to mind). (2) Abduction involved re-grouping (cat was exonerated and relation (cat *pushed* vase) was de-established. Instead, relations (child *kicks* ball, and ball *pushes* vase) were formed). 

#### 5.2.8. Dynamics of Thinking

Thinking is predicated on stability of memory structures and reversibility of cognitive operations [7], demanding minimization of entropy in the system. At the same time, exploration of the system’s phase space and identification of instabilities require injections of entropy [16]. This proposal suggests that temperature variations provide the requisite injections, causing the system to pulsate between far-from-equilibrium and equilibrium states (which might correlate roughly with the experience of alternations between effortful attention focusing and diffuse attention, and spontaneous associative shifts). Consistent with the present theory, recent approaches in the analysis of brain processes associate transient dynamics with information production [136]. Conceivably, neuronal avalanches [137] underpin alternations between the states, helping to satisfy the competing demands of information transmission and network stability.

#### 5.2.9. Learning with and without Understanding

The distinction is best explicated using the notions of *fluid* and *crystallized* intelligence [138]. Roughly, the latter term denotes ability to learn and to act based on the results of learning. By contrast, the former term denotes the ability to deviate from the directives of prior learning and to act adequately under unfamiliar conditions. With the present theory, *fluid intelligence* is predicated on understanding and builds on top of *crystallized intelligence*. More technically, learning involves synaptic modifications represented in a mosaic of link weights in the associative network. Packets form in the associative networks but their formation, grouping into models, and operations on models leave the weight mosaic intact. Understanding capacity was a product of evolutionary development building on the learning capacity, and served to overcome its limitations (e.g., cats are often observed attacking small moving objects (large associative weights) and hardly ever observed attacking large stationary objects (small weights). As a result, rigid reliance on a *crystallized* weight mosaic would have precluded the “cat *pushed* vase” idea, leaving the objects uncoordinated in the subject’s mind and rendering the scene unexplainable). 

#### 5.2.10. Meaning and value

Meaning of information is determined by the mental model where the information is fitted in. For example, in the “broken vase” test, a hint informing the subject that “a child was playing with a ball nearby” would make sense if the subject was able to grasp the relation and would remain meaningless if otherwise. Value is a function of worth attributed to the objects and the outcomes of modeling (accordingly, meaningless information has no value). 

#### 5.2.11. Neuroenergetics

Most brain energy is used on synapses [139,140] This theory itemizes the account by introducing costs incurred in the navigation of the energy landscape in which the synaptic network is embedded. It has been demonstrated that pre- and postsynaptic terminals in the neurons are optimized to allow maximum information transmission between synapses at minimal energy costs [141]. This theory contends that: (a) the brain’s functional architecture is optimized to allow maximum information production at minimal energy costs, (b) optimization involves mechanisms controlling the interplay between the costs of engagement (exciting/inhibiting neurons) and the costs of navigation, and (c) the understanding capacity is a product of such optimization. 

#### 5.2.12. Gnostron

Gnostron framework combines elements of reinforcement and unsupervised learning in the formation of packet networks, and admits the use of other techniques in network processing (e.g., Bayesian updating, probabilistic inter-packet routing [142], other). Gnostron process can be viewed as a form of mapping different from that used in perceptron: perceptron (neural nets) seeks to establish mapping between vectors while gnostron seeks to establish coordination between patterns of vector movement. Establishing coordination involves combining packets into models, which underlies understanding and attainment of meaning. In short, perceptron learns to recognize conditions while gnostron learns to understand them. Technically, gnostron is an adaptive controller obtaining progressively improving efficiency via operations on self-organizing vector spaces [143]. On the present theory, gnostron implements the key function attributed to the human brain: transforming energy into the work of information production

### 5.3. Further Research—A Fork in the Road

This proposal seeks to form a conceptual bridge between two foundational ideas in neuroscience: the idea of neuronal assembly [34,35] and the idea of Markov blanket and variational free energy minimization [10,11]. In a sense, these ideas reside in a two-dimensional space defined by neuropsychological and information-theoretic axes. The bridging idea (neuronal packets) positioned energy (thermodynamics) as the third dimension. The intuition was that energy processes are not alien and external to the cognitive machinery (e.g., a horse is alien and external to the cart it pulls) but are interwoven into it at every level [30,31]. Suggestions resonating with this idea are now beginning to enter the mainstream from many directions (e.g., energy-aware computing [144]), in a radical departure from the conventional AI and cognitive science framework. Developments along these new lines quickly arrive at a “fork in the road”, posed by some of the most entangled and challenging issues in science—the role of the second law in biophysics and physical underpinnings of information processing. The two paths in the fork are determined by the way the operation of the second law in the development of life and cognition is conceptualized. Both paths assume that the second law drives optimization in the cognitive system but the choices of the optimization criteria could not be more different: (A) Cognitive machinery is optimized to maximize entropy production, or (B) cognitive machinery is optimized to maximize information production.

(A) The notion that evolution selects for maximum entropy production derives from the assumption that “order produces entropy faster than disorder” [145,146]. With this notion, proliferation of forms and progression from simple to more richly-structured forms are manifestations of “zigzags” between low entropy pockets (complex forms), executed by nature in its rush downhill, towards universal homogeneity and dissolution of all forms (by implication, growing order entails accelerated descent). The following comments question not the assumption but its usefulness in the study of cognition. A caricature analogy of the assumption would be equating the role of digestion to production of waste. With that, a measure of digestive efficiency would be the ratio of the amount consumed to the amount expelled, overlooking extraction of nutrients and their role in keeping the organism alive. One can accept that evolution (say, from protohuman to Sapience) was accompanied by increased entropy production in the brain counteracted by accelerated entropy removal. Even if proven correct, the result would not shed much light on the mechanisms of cognition. The Internet can be viewed as a means of information processing or as a drain on resources. As in the Necker cube, both perspectives are possible but one of them opens a view on search engines, while the other one obstructs it. The formula “from dust to dust” is undoubtedly correct but short circuits enquiries into what might be happening during transit. In short, the entropy maximization principle can hardly inform analysis of cognition or design of intelligent artifacts. 

(B) Cognition involves information production predicated on entropy reduction in the neuronal system. As formulated by Konrad Lorentz:

“Without offending against the principle of entropy in the physical sense … all organic creation achieves something that runs exactly counter to the purely probabilistic process in the inorganic realm. The organic world is constantly involved in a process of conversion from the more probable to the generally more improbable by continuously giving rise to higher, more complex sates of harmony from lower, simpler grades of organization”.

[147]

Both (A) and (B) agree on the vector of evolution (from the simple to the complex) but disagree on the assessment of where the vector points: (a) the organic world rushes itself downhill towards self-destruction, or (b) the organic world pushes itself uphill towards self-comprehension, culminating in the development of the understanding capacity. 

The latter viewpoint suggests the following lines of enquiry:

**(1) Cognitive thermodynamics.** Statistical thermodynamics (thermal physics) addresses energy processes in simple systems (e.g., ideal gas, inorganic compounds) [148]. Biological thermodynamics addresses energy transformation in the living matter [149,150]. Cognitive thermodynamics focuses on the energy processes in the nervous system that underpin cognitive functions, seeking to integrate various theoretical constructs (metastability and phase transition in the brain [151,152], cortical coordination dynamics [153], neuronal group selection [154], dynamical systems [155,156], self-organization in the brain, [157], embodied cognition [158], other) within a unifying framework defined by the Markov blanket and free energy minimization principles. The following conjectures are within the scope of this enquiry. Mental modeling creates mechanisms amplifying thermodynamic efficiency of neuronal processes in the volume of the model, including:

- Converting excessive heat into work;
- Biasing ATP hydrolysis towards accelerating release of Gibbs free energy and inhibiting release of metabolic heat;
- Reducing Landauer’s cost of information processing (by regulating access in the landscape).

**(2) Neuropsychology of understanding.** Reducing cognition to possession of disembodied algorithms entailed excising understanding capacity from the purview of cognitive theory.

“Unified theories of cognition are single sets of mechanisms that cover all of cognition—problem solving, decision making, routine action, memory, learning, skill, perception, motor activity, language, motivation, emotion, imagining, dreaming, daydreaming, etc. Cognition must be taken broadly, to include perception and motor activity”.

[159]

(please recollect that understanding was presumed to play a marginal role in problems solving, if any [104], as seen Section 5.1.) Neuropsychological theory of understanding has a dual objective of: (a) analyzing performance benefits conferred by the understanding capacity, and (b) elucidating the underlying neuronal mechanisms, aiming at representing them within a unified functional architecture (architecture for understanding) (e.g., contingent on further analysis, the architecture might account for recent findings indicating that processing of plausible and implausible data engages different pathways in the brain [160]). The theory needs to be broad enough to allow comprehensive analysis of the role played by understanding in different manifestations of intelligence (“multiple intelligences” [161]). 

**(3) Machine understanding.** Machine intelligence builds on the results of the above enquiries, implementing a transition from machine learning (knowledge-based systems) to machine understanding (understanding-based systems). Understanding-based systems combine energy efficiency with the ability to construct adequate responses under unforeseen and disruptive conditions, and to explain decisions motivating the responses. Construction derives response elements and their organization (procedures, algorithms) from internal models; explanation capabilities are organic to the system, accounting for operations on models that are inherently intelligible (e.g., grasping relations) and intrinsic to the decision process. Such systems can act autonomously or collaboratively, predicating their overt actions on the results of self-assessment seeking to verify that understanding of the task and circumstances is sufficient for executing the task. 

### 5.4. Digest

Life emerges in molecular networks, when subnets fold into quasi-stable aggregations bounded by surfaces (Markov blankets), conferring a degree of statistical independence to the internals. Sustaining life requires regulating flows of matter and energy through the boundary surface. Folding in networks appears to be the mechanism used in both creating life and regulating life; subnets in neural networks fold into quasi-stable aggregations (neuronal packets) bounded by energy barriers. Matching such packets against changing conditions (stimuli stream) at the organism’s boundary surface creates packet networks reflecting order (regularities) in the stream. The process is stimuli-driven, thus amounting to absorbing information and extracting negentropy from the stream. The tendency to improve matching scores (minimize surprise) gives rise to processes operating on packet networks and combining packets into new structures (models). Operations on models are decoupled from the stimuli stream and self-directed, thus amounting to information production and negentropy generation. Modeling prepares the system to future conditions, thus radically improving matching scores and giving rise to the experience of attaining understanding. Modeling processes are governed by an interplay of two criteria: improving the scores and reducing overhead (energy costs incurred during modeling). The interplay makes the system self-aware and motivates continuing construction, modification, and integration of models, in a spiral of information production and growing understanding. The takeaway notion concerns distinctions between learning and understanding, as follows. Learning allows extrapolation, i.e., draws a line connecting the past and the present and extends it into the future. Mental modeling allows the extended line to be split into a bundle (what-ifs). Understanding employs a form of modeling that submits for attentive examination a few lines in the bundle plausible under the multitude of factors impinging on the outcomes of interest. Understanding does not foretell the future but accounts for the past, explains the present, and offers the lowest ceiling on future surprises.
